# Extracellular Vesicles of Probiotics: Shedding Light on the Biological Activity and Future Applications

**DOI:** 10.3390/pharmaceutics15020522

**Published:** 2023-02-04

**Authors:** Paweł Krzyżek, Beatrice Marinacci, Irene Vitale, Rossella Grande

**Affiliations:** 1 Department of Microbiology, Faculty of Medicine, Wroclaw Medical University, 50-368 Wroclaw, Poland; 2 Department of Pharmacy, University “G. d’Annunzio” of Chieti-Pescara, Via dei Vestini, 31, 66100 Chieti, Italy; 3 Department of Innovative Technologies in Medicine & Dentistry, University “Gabriele d’Annunzio”, Chieti-Pescara, 66100 Chieti, Italy

**Keywords:** extracellular vesicles, membrane vesicles, probiotics, probiotic bacteria, postbiotics

## Abstract

For many decades, the proper functioning of the human body has become a leading scientific topic. In the course of numerous experiments, a striking impact of probiotics on the human body has been documented, including maintaining the physiological balance of endogenous microorganisms, regulating the functioning of the immune system, enhancing the digestive properties of the host, and preventing or alleviating the course of many diseases. Recent research, especially from the last decade, shows that this health-benefiting activity of probiotics is largely conditioned by the production of extracellular vesicles. Although the importance of extracellular vesicles in the virulence of many live-threatening pathogens is widely described in the literature, much less is known with respect to the health-promoting effect of extracellular vesicles secreted by non-pathogenic microorganisms, including probiotics. Based on this, in the current review article, we decided to collect the latest literature data on the health-inducing properties of extracellular vesicles secreted by probiotics. The characteristics of probiotics’ extracellular vesicles will be extended by the description of their physicochemical properties and the proteome in connection with the biological activities exhibited by these structures.

## 1. Introduction

For time immemorial, the proper functioning of the human body has become a leading topic undertaken by scientists [1]. It quickly became clear that there is a very strong relationship between human health and its resident microbiota [2,3]. As a resultant, the idea of using probiotics was created, i.e., live microorganisms that, when administered in the proper dose, have a beneficial effect on the host [1,4]. One of the first well-documented example of the usefulness of probiotics dates back to 1907, from the observations made by Elie Metchnikoff, who showed the existence of a positive correlation between the consumption of fermented food containing probiotics and the lifespan of the Bulgarian population [1]. This was the starting point for further observations of health-benefiting properties of this microbial group and the development of a powerful trend of research on probiotics in the following years [1,5]. After several decades of experiments, a striking impact of probiotics on the human body has been documented, including maintaining the physiological balance of microbiota, regulating the functioning of the immune system, enhancing the digestive properties of the host, and preventing or alleviating the course of many diseases [1,5,6]. The growing awareness of the benefits of probiotics has contributed to the exponential growth of commercial products containing these microorganisms [4]. The use of probiotics has undoubtedly become very popular, however, their viability in such preparations is often questioned, which is related to their exposure to various unfavorable parameters, such as processing (e.g., dehydration), storage conditions, and physiology of the product’s target site (e.g., passage through the physicochemically diverse, harsh environment of the digestive system) [7].

Although probiotics are currently in the center of interest of pharmaceutical concerns, more and more research focuses on searching for alternatives to these classically used products [8,9]. Difficulties in maintaining the viability of probiotics in commercial products and the fact that the viability of these microorganisms is not always necessary to obtain therapeutic effects have led to the concept of postbiotics [5,7,10,11]. Postbiotics are a mixture of metabolic products or non-viable fragments of probiotics that have a beneficial effect on the functioning of the human body [8,12]. Despite the fact that research on postbiotics is still in its infancy, a number of health-promoting properties of these new products have already been demonstrated (maintenance of the proper structure of the resident microbiota, strengthening the host epithelial barrier, modulation of the local and systemic immune response, or increase of the host metabolic activity) [12]. As non-viable elements of probiotics, postbiotics present their strictly defined technological properties and thus represent a promising tool for obtaining therapeutic effects [10,11,13]. These parameters include a favorable level of absorption and distribution of postbiotics [13] and the lack of risk to spread resistance mechanisms, as documented between probiotics and microbiota or pathogens [12]. In 2021, the International Scientific Association of Probiotics and Prebiotics established a new definition of postbiotic as a “preparation of inanimate microorganisms and/or their components that confers a health benefit on the host” [14]. The use of the word ‘components’ was made because the whole microbial cells are not always required to present health-promoting activity and such an impact may be related to the presence of cellular structures produced by probiotics. The above modification of the meaning of the term ‘postbiotic’ has opened a new avenue for many new categories of these preparations, including probiotic extracellular vesicles [12].

Extracellular vesicles (EVs) are nanoscale lipid particles secreted by virtually every type of living cell [15]. For this reason, the use of various nomenclature in the description of these structures is frequent. This richness in the nomenclature of the vesicles is related to their physical properties (mostly size), the way they are formed, or the cells that secrete them [15,16,17]. In order to standardize the nomenclature as much as possible, in accordance with the recommendations of the International Society for Extracellular Vesicles [18], in this review, we will use the term “extracellular vesicles”. This is a collective term classically referring to various types of small compartments released from cells, which are surrounded by a lipid bilayer and incapable of spontaneous replication [18]. The main function of EVs is the transport of various classes of macromolecules, including lipids, polysaccharides, proteins, and nucleic acids [9,16,19]. Through this cargo, these structures perform a number of key functions in microorganisms, including long-distance transport of nutrients, protection against environmental stressors, or communication during microorganism–microorganism or microorganism–host interactions [16,17,20]. The importance of EVs in the virulence of many live-threatening pathogens is well known and widely described in the literature [21,22]. Much less is reported with respect to the health-promoting effect of EVs secreted by non-pathogenic microorganisms, including probiotics. For the last few years, however, awareness on probiotic EVs as a very promising therapeutic platform is growing rapidly [15,17,19]. This seems to be strictly related with both the ability of EVs to carry many different bioactive macromolecules and with nanometric dimensions of these structures.

Based on the above facts, in this review article, we decided to collect the latest literature data on the health-promoting properties of extracellular vesicles secreted by probiotics. These characteristics will be extended by the description of physicochemical properties and the proteome of EVs produced by probiotic microorganisms.

## 2. Review Strategy and Literature Included

To obtain articles comprising the central core of the current review paper, we used the Scopus and PUBMED databases. In that respect, only English-language original articles from the last decade (1 January 2012–30 June 2022) were included. The search terms were “membrane vesicles” or “extracellular vesicles” together with “probiotics”. In order to obtain as many records as possible, an additional search phase was performed involving the use of “membrane vesicles” or “extracellular vesicles” together with different genera of the most important probiotic microorganisms, e.g., “*Lactobacillus*” (and all new genera from the Lactobacillaceae family), “*Bifidobacterium*”, “*Lactococcus”*, “*Pediococcus*”, “*Propionibacterium*”, etc. As a result of this, we were finally able to obtain 73 original articles, which were subjected to our further analysis and description in subsequent parts of the current review.

## 3. Discussion

### 3.1. Physicochemical Properties of EVs Produced by Probiotics

**Figure 1:**
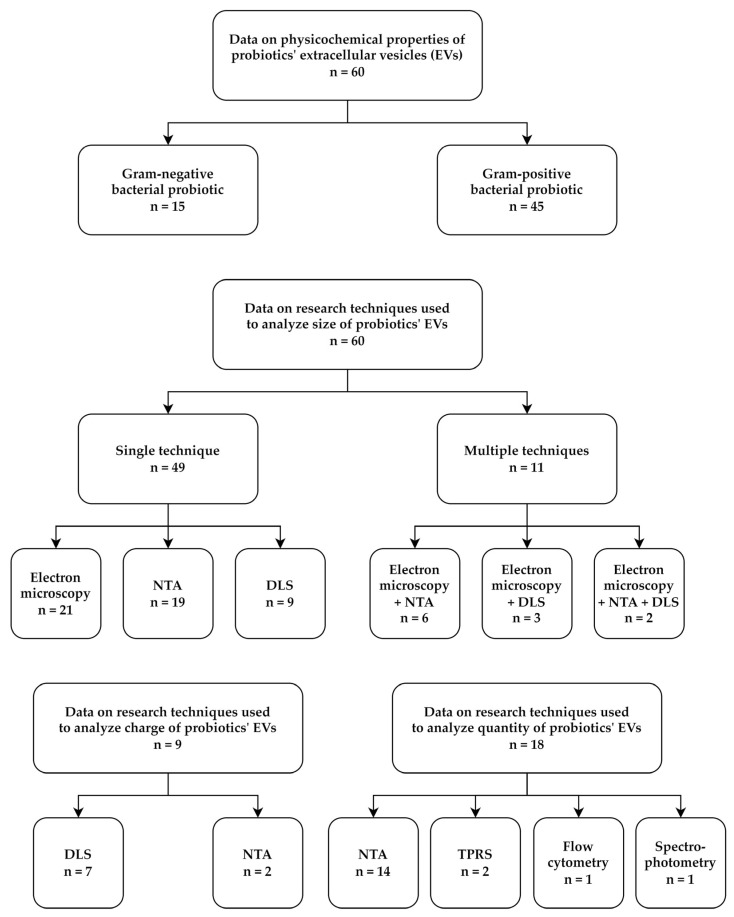
A graph presenting the categorization of articles constituting the core of this review, in which the physicochemical properties of extracellular vesicles (EVs) of probiotics were described. Among the 60 of them, 15 and 45 concerned Gram-negative and Gram-positive bacteria, respectively. All articles assessed the size of the produced EVs, of which 49 determined it using only one technique (21 with electron microscopy [51,52,53,54,55,56,57,58,59,60,61,62,63,64,65,66,67,68,69,70,71], 19 with NTA [72,73,74,75,76,77,78,79,80,81,82,83,84,85,86,87,88,89,90], and 9 with DLS [42,45,49,50,91,92,93,94,95]), while 11 used more than one technique (6 with electron microscopy + NTA [29,30,31,32,33,34], 3 with electron microscopy + DLS [35,36,37], and 3 with electron microscopy + NTA + DLS [38,39]). Only 9 papers analyzed the electrical charge of EVs, of which 7 used DLS [31,36,37,38,39,42,45] and 2 used NTA [84,85]. The quantity of EVs produced was examined in 18 articles, of which 14 used NTA [31,33,38,39,72,73,74,76,77,79,83,87,88,89], 2 used TRSP [49,50], 1 used flow cytometry [45], and 1 used spectrophotometry [51]. Detailed information on the numerical data of the above-presented results can be found in Table S1. Abbreviations: DLS, dynamic light scattering; NTA, nanoparticle tracking analysis; TRSP, tunable resistive pulse sensing.

Our review of the literature on the ability of probiotic bacteria to produce EVs began with the collection of data describing EVs’ physicochemical properties (Table S1). We noticed that this information was available for 60 out of the 73 publications being the central core of this review. Interestingly, in 75% of the cases (45/60), the results concerned Gram-positive bacteria, in particular from the Lactobacillaceae family (Figure 1 and Table S1). The group of Gram-negative bacteria consisted of only two representatives—*Escherichia coli* Nissle 1917 and *Akkermansia muciniphila* ATCC BAA-835 (Figure 1 and Table S1). In our opinion, this situation is the result of frequent interchangeable use of two words with different definitions—‘probiotics’ and ‘lactic acid bacteria (LAB)’ [23,24]. The term ‘probiotics’ is broader and refers to many different groups of microorganisms with beneficial properties for the host, while the term ‘LAB’ refers only to Gram-positive, lactic acid-producing bacteria for which, many decades ago, numerous health-promoting properties were proved, and, hence, these bacteria were quickly classified as the most important representatives of probiotics [23,24,25,26]. In recent years, a strong separation between these two terms is made, contributing to the gradual expansion of research on probiotic properties of Gram-negative bacteria and their EVs. In this context, a mucin-degrading, Gram-negative bacterium *A. muciniphila* deserves special attention, as it was isolated for the first time as lately as in 2004 [27], while, in recent years, there has been an undoubted bloom in interest in this bacterium and its EVs [28]. In our opinion, the above situation has a chance to encourage other researchers focusing on the subject of probiotic microorganisms to expand their search with new, valuable species of probiotics from the group of Gram-negative bacteria.

A careful analysis of the data collected by us showed that, in all 60 articles, the dimensions of probiotics’ EVs were determined, while, for this purpose, various research techniques were used (Figure 1 and Table S1). The most frequently applied methods were electron microscopy (21/60; 35%) or nanoparticle tracking analysis (NTA) (19/60; 31.7%). Dynamic light scattering (DLS) (9/60; 15%) was used less commonly for this purpose (Figure 1 and Table S1). In 11 articles, more than one technique was applied to determine the size of EVs, including 6 with microscopy + NTA [29,30,31,32,33,34], 3 with microscopy + DLS [35,36,37], and 2 with microscopy + NTA + DLS [38,39] (Figure 1 and Table S1). Among other parameters of EVs measured by researchers, the surface charge/zeta potential and the quantity of EVs can be distinguished. In both cases, the subject was, however, undertaken relatively rarely, i.e., 9/60 (15%) and 18/60 (30%), respectively (Figure 1 and Table S1). The surface charge of EVs was detected most often with the use of DLS (7 of 9 articles) and the quantity of EVs with the use of NTA (14 of 18 articles).

Looking closer at the size analysis of probiotics’ EVs, we noticed a large spectrum of results, which depended on both the tested strains/species/genera of bacteria and the analytical techniques used. For Gram-negative bacteria, most of the dimensions were in the range of 20–200 nm (Table S1). On the other hand, for Gram-positive bacteria, the range of the obtained results was greater, although often equal to 50–300 nm (on average, approx. 150 nm) (Table S1). In a comparative context, the articles in which the dimensions of EVs were measured using two or three techniques seem particularly valuable. For example, Hu et al. [29], Liu et al. [30], and Müller et al. [31] showed the convergence of EVs’ size values between electron microscopy and NTA, in which the former showed a wider range of detected sizes, while the latter often narrowed this range down to specific values (Table S1). In line with this, electron microscopy might be better than NTA in the analysis of EVs with very low dimensions, as the former suffers from sensitivity and resolution limitations [40,41]. On the other hand, in contrast to NTA, using electron microscopy, it is difficult to precisely determine the mean size of EVs (often being presented as a range of sizes) and it is impossible to determine the concentration of EVs in the sample [40,41]. Nevertheless, the usefulness of both techniques is reflected in the frequency of their use in the articles we analyzed (in total, 51 out of the 60 cases applied one or both of them) (Figure 1 and Table S1). In the context of DLS, according to Shao et al. [40], this method seems also to be quite useful in measuring the dimensions of EVs, but it should be remembered that the critical step during the analysis is to use number distribution, as original size distribution is intensity-weighted and large EVs may over-dominate the obtained results.

As mentioned previously, the parameters of EVs other than dimensions—surface charge/zeta potential and quantity—are of little interest to scientists. In the studies in which this topic was addressed, the zeta potential of EVs had negative values between −0.5 mV [37] and −45 mV [42], with an average ranging from −10 mV to −20 mV (Table S1). The phenomenon of negative charge of EVs is most often related to the presence of extracellular DNA on the surface of these structures, which translates into their important function in supporting adhesion, aggregation, and biofilm formation of microbes [43,44]. Due to this, the electric charge of the EVs of probiotic bacteria can directly affect their colonization capacity of the host [31,39,45]. It seems to us that the low interest in this parameter among the scientific community can be explained by the willingness to administer to patients purified EVs of probiotics, without applying microorganisms secreting them (e.g., in a form of postbiotics) [46]. Although the influence of EVs on the colonization capacity of the probiotics producing them is not widely investigated, it should still be kept in mind that EVs applied in this way could affect the diversity and properties of the host microbiota [47,48].

Taking into account the amount of EVs produced by probiotics, these values were in the range of 10^8^–10^12^ per mL when focusing on the most commonly used technique—NTA (14/18; 77.8%) (Table S1). For the other two techniques, tunable resistive pulse sensing (TRPS) [49,50] and flow cytometry [45], these values were equal to approximately 10^10^ and 10^7^ per mL, respectively (Table S1). For spectrophotometry [51], the unit used (relative fluorescence units/colony forming units of probiotics) makes it impossible to compare the results with the others (Table S1). Although the frequency in measuring quantity of probiotic EVs was quite low, the high homogeneity of applied research techniques allows for a relatively objective comparison of EVs’ production by different probiotics. In our opinion, the lack of universality in measuring the efficiency of production of probiotic EVs is, as stated before, associated with the frequent perception of these EVs as a therapeutic agent with designation to be administered in a purified form to patients (as postbiotics) and not necessarily as structures that would be secreted by the probiotic into the local environment, e.g., the intestines [46].

Other aspects of the biological and physicochemical properties of EVs secreted by probiotics, including the spatial orientation of EVs’ membranes or their biological origin (including the participation or lack of cell lysis), were examined extremely rarely—only in the case of single original articles. Therefore, the above properties were not included in the main part of our discussion. The description of the aforementioned problem will be additionally deliberated in Section 3.5. “Challenges and Limitations of Articles Focusing on EVs Produced by Probiotics”.

### 3.2. Proteomic Profile of EVs Produced by Probiotics

The second aspect analyzed by us was the evaluation of the proteome of EVs produced by probiotic bacteria. Out of 73 articles constituting the core of this review, 17 took up this topic (Table 1). Since the methodology of isolation and purification of EVs or analysis of their proteins may influence the obtained results, we decided to collect this information in Table 1. We noticed that, in both cases, there is a relatively high homogeneity of the research techniques used. Most probiotics’ EVs were isolated by ultracentrifugation (14/17; 82.4%), while, in the remaining cases, chemical precipitation (1/17) or size exclusion chromatography (SEC) (2/17) was applied (Table 1). In the analysis of the EVs’ proteome, the most frequently used technique was electrophoresis combined with liquid chromatography with tandem mass spectrometry (LC-MS/MS) or matrix-assisted laser desorption/ionization-time of flight (MALDI-TOF) (12/17; 70.6%). Less frequently, chemical (4/17) or magnetic (1/17) precipitation combined with LC-MS/MS was used for this purpose (Table 1).

Despite the relatively high methodological homogeneity, a large discrepancy within the data was noticed. The total number of identified proteins (understood as whole-length gene products) ranged from a dozen [74] to over a thousand (1149 [54] and 1283 [62]), while, in most articles, these values were within the range of several hundred (10/17; 58.8%) (Table 1). In this context, it is also worth highlighting the strong correlation between cellular localization and the number of isolated EVs’ proteins. If the protein localization was classified as ‘membrane’, then the proteome was narrower (11–192 proteins) than when the most abundant protein representation was derived from the cytoplasm (11–1286 proteins; in all cases where the proteome was >300 proteins, ‘cytoplasm’ dominated) (Table 1). This phenomenon may be caused by two sources—the sensitivity of the research techniques and the level of contamination of the EVs’ proteome with proteins derived from bacterial cells producing these structures [40,96,97]. Insufficient level of sensitivity may contribute to the loss of proteins with low representation in the EVs’ proteome, the function of which, however, may be of key importance for microorganisms secreting them [98,99]. On the other hand, too high representation of proteins in the EVs’ proteome may suggest its contamination and the need to include/improve the purification step of the obtained EVs [96,100]. According to the review by Nagakubo et al. [96], some researchers consider numerous representations of ribosomal proteins (30S and 50S), which are typically of cytoplasmic origin, as an independent indicator of the EVs’ proteome contamination. However, there are articles showing that extracellular secretion of ribosomal proteins may have important, extra-ribosomal functions for the physiology of microorganisms, including biofilm formation [101] or resistance against translation-targeting antibiotics [102,103]. Therefore, in our opinion, the detection of ribosomal proteins in the EVs’ proteome should not be automatically interpreted as contamination, however, serious consideration for improving the techniques of isolation and analysis of EVs should be made if the proteome of these structures is both too numerous and over-represented by cytoplasmic proteins, including, in particular, the ribosome subunits.

In addition to information about the methodology of isolation of EVs and analysis of their proteome, in Table 1 we also included some details about the most abundant/most important proteins constituting the EVs’ proteome of probiotics. To simplify this issue, once again, we have decided to divide the discussion into a part covering Gram-negative and Gram-positive bacteria.

Gram-negative bacteria described in Table 1 were represented only by *E. coli* Nissle 1917, while as many as 4 out of the 17 articles focused on this aspect [29,51,52,72]. It is worth noting that within the most numerous proteins secreted by this bacterium in EVs many adhesive proteins were highlighted, including fimbrial (FocA, Fim1C, FocF, FocG, and FocH) and flagellar (FliC, FliD, FlgA, FlgE, FlgK, and FlgL) subunits, and outer membrane proteins (OmpA, OmpC, OmpF, and NmpC) (Table 1). According to many, the presence of adhesins anchored on the surface of EVs of Gram-negative bacteria is an important element facilitating the colonization of the intestines [104,105,106]. The second important group of proteins produced by this bacterium within EVs was related to peptidoglycan and cell membrane rearrangement, i.e., murein hydrolases (MltA, MltB, and MltC), murein-interacting protein MipA, and peptidoglycan-associated lipoproteins (Pal, TolB, Sat, LpoA, YbaY, and SlyB) (Table 1). It is indicated that the rearrangement of murein and cell membranes are important steps in the biogenesis of EVs, hence, the presence of proteins related to the above processes within EVs should not come as a surprise [107]. On the other hand, researchers pay attention to the participation of murein hydrolases encased within EVs in the competitive fight against other bacteria [108]. In summary, the main components of the EVs’ proteome of *E. coli* Nissle 1917 were adhesins and proteins associated with peptidoglycan rearrangement, which are involved in the effective colonization of the host and its protection against pathogenic microorganisms.

Gram-positive bacteria described in Table 1 were mainly representatives of the Lactobacillaceae family (*Lactiplantibacillus, Lacticaseibacillus, Limosilactobacillus, Ligilactobacillus,* and *Lactobacillus*), and, additionally, by *Bifidobacterium, Propionibacterium, Lactococcus,* and *Pediococcus*. Among the dominant group of proteins located within EVs of these bacteria were metabolic proteins, while, in single cases, peptidoglycan rearrangement proteins (including putative murein hydrolases or lysozyme-like proteins [32,34,36,38]) and proteins related to adhesion or aggregation (including surface proteins, mucus-binding proteins, or aggregation-promoting factors [33,38,78,79]) were also noticed (Table 1). Whereas the function of adhesins and peptidoglycan rearrangement proteins in EVs has been described above and is similar in Gram-positive bacteria, proteins responsible for metabolism deserve special attention. Based on the data collected in Table 1, it can be noticed that this group includes proteins related to the biosynthesis or breakdown of various classes of nutrients, and they are produced by many representatives of probiotics, such as *Lactiplantibacillus* [32,54], *Lacticaseibacillus* [36], *Limosilactobacillus* [34,38], *Ligilactobacillus* [76], *Lactobacillus* [33,74], *Bifidobacterium* [64], *Propionibacterium* [78,79], *Lactococcus* [62], and *Pediococcus* [42]. In addition to this, numerous proteins determining an uptake of glycerol [54], vitamins [54], amino acids and peptides [54,76], phosphates [74,76], inorganic acid ions [76], and iron [64] were detected. All the above-mentioned proteins participate in the transport of nutrients from the local environment and their delivery in an assimilable form. Many scientists point out that this system can provide nutrients not only to the EVs-producing microorganisms, but also to the host, especially in areas with high availability of nutrients, e.g., intestines [109,110,111]. On this basis, it can be concluded that EVs of Gram-positive probiotic bacteria determine not only their colonization abilities and competition with pathogens, but also may improve digestive processes of the host.

As it can be easily observed, EVs produced by probiotics surely have a different role in the host than those released by pathogens. With regards to probiotics, we speculate that the role of EVs secreted by different probiotics is strain-dependent and, in fact, influenced by the bacterial phenotype, culture conditions (i.e., culture media used or the age of microbial culture), and biogenesis mechanisms by which such EVs are released—including differences between Gram-positive and Gram-negative species [112,113]. For this reason, currently, we cannot state that a “universal molecular mechanism” conditioning benefit of probiotic EVs for the host exists. Unquestionably, further studies based on a deeper proteomic and metabolomic analysis of probiotic EVs, which compares different microbial strains and culture conditions, should be performed to discover microbial components capable of conferring beneficial properties on the host.

**Table 1:** Proteomic data on extracellular vesicles produced by probiotics.

Bacterial Producer	Methodology of Isolation/Determination	Proteomic Data			Reference
		Total Number of Identified Proteins *	Cellular Localization of Proteins	The Most Abundant Representatives/ Proteins Highlighted by Authors	
*Escherichia coli* Nissle 1917	Ultracentrifugation (vesicles) Electrophoresis + LC-MS/MS (proteome)	192	Outer membrane ~ 40% Cytoplasm 40% Periplasm 15% Inner membrane < 5 %	- Fimbriae subunits: FocA, FocF, FocG, FocH - Flagellins: FlgK, FlgE, FliD - Murein hydrolase MltB - Metabolic proteins: AnsB, CadA, FbaB, GapA, Icd, Mdh	[52]
	Ultracentrifugation + DGC (vesicles) Electrophoresis + LC-MS/MS (proteome)	189	Outer membrane 28% Cytoplasm 36.5% Periplasm 20% Inner membrane 9.5% Secretory 6%	- Fimbriae subunits: FocA, FocF, FocG, FocH - Flagellins: FlgA, FlgE, FlgK - Outer membrane proteins: OmpA, OmpC, OmpF - Murein hydrolases: MltA, MltC	[29]
	Ultracentrifugation (vesicles) Electrophoresis + LC-MS/MS (proteome)	295	*ND*	- Peptidoglycan-associated lipoproteins: Pal, TolB - Murein-interacting protein MipA - Flagellin subunit FliC - Outer membrane proteins: OmpA, OmpC, OmpF, NmpC	[51]
	Ultracentrifugation + DGC or SEC (vesicles) Chemical precipitation + LC-MS/MS (proteome)	189	Membrane ~ 60% Cytoplasm ~ 40%	- Peptidoglycan-associated lipoproteins: Pal, Sat, LpoA, YbaY, SlyB - Fimbrial protein Fim1C - Flagellin FlgL - Outer membrane proteins: OmpC, NmpC	[72]
*Lactiplantibacillus plantarum* BGAN8	Ultracentrifugation (vesicles) Electrophoresis + MALDI-TOF (proteome)	1149	Membrane ~ 45% Cytoplasm + ribosomes ~ 52% Secretory < 1%	- Polysaccharide biosynthesis proteins: EpsN, MurJ - Transporters of glycerol (GlpF), niacin (NiaP), oligopeptides (OppC), amino acids (SdaC, CycA) - Translation proteins: 30S and 50S ribosomal subunits (55 different subunits)	[54]
*Lactiplantibacillus plantarum* WCFS1	Chemical precipitation (vesicles) Electrophoresis + LC-MS/MS (proteome)	31	Membrane 42% Cytoplasm 13% Secretory 16%	- Cell wall remodeling proteins: Acm2, DltD, MreC, Lp_2847, Lp_3015, Lp_2162, Lp_3093, Lp_3421 - Metabolic proteins: TpiA, GapB, Pgi, Ldh1	[32]
*Lacticaseibacillus casei* ATCC 393	Ultracentrifugation (vesicles) Chemical precipitation + LC-MS/MS (proteome)	43	Membrane ~ 20% Cytoplasm 65% Secretory 14%	- Putative family 15 glucoamylase LBCZ_2692 - Lysozyme-like proteins: LBCZ_0210, LCAUW4_1864 - N-acetylmuramoyl-L-alanine amidase LSEI_1536 - Putative cell wall-associated hydrolase LSEI_0281	[38]
*Lacticaseibacillus casei* BL23	Ultracentrifugation (vesicles) Electrophoresis + LC-MS/MS (proteome)	103	Membrane + secretory 43% Cytoplasm 57%	- Cell wall-associated hydrolases P40 and P75 - Metabolism proteins: GapA, Pgk, LdhL, Fba, AspB, Pyk - Translation proteins: 30S ribosomal subunits (RpsJ, RpsL, RpsS, RpsT, RpsU) and 50S ribosomal subunits (11 different subunits)	[36]
*Limosilactobacillus reuteri* ATCC 23272	Ultracentrifugation (vesicles) Chemical precipitation + LC-MS/MS (proteome)	17	Membrane 18% Cytoplasm 82%	- Metabolic proteins: Lreu_0426, Lreu_1721, Lreu_1853 - NAD kinase NadK	[38]
*Limosilactobacillus reuteri* BBC3	Ultracentrifugation + DGC (vesicles) Chemical precipitation + LC-MS/MS (proteome)	92	Membrane 27% Cytoplasm 56.5% Secretory 16%	- Metabolic proteins: GatB, ProS, SerS, IleS, LRI_0925, B5F04_03325, N134_06765 - Cell wall remodeling proteins: Lr1610, MreC - Translation proteins: 30S ribosome subunit (RpsB) and 50S ribosome subunit (RplC)	[34]
*Ligilactobacillus animalis* ATCC 35046	Ultracentrifugation + DGC (vesicles) Chemical precipitation + nLC-MS/MS (proteome)	340	From the top 74 proteins: Membrane 25.7% Cytoplasm 25.7% Secretory 1.3% Unknown 47.3%	- Sortase SrtA - Transporters of phosphate (PstS), glutamine (GlnP), nitrate/sulfonate/bicarbonate (Lani381_1252) - Metabolism protein Pgi - Antioxidative protein Dsp	[76]
*Lactobacillus acidophilus* ATCC 53544	Ultracentrifugation (vesicles) Chemical precipitation + LC-MS/MS (proteome)	26	Membrane ~ 30% Cytoplasm 62% Secretory 12%	- Surface proteins: FmtB, SlpX - Mucus binding protein Mub - Transporters of maltose (LBA1864) and glutamine (GlnP) - Bacteriocin LBA1805	[38]
*Lactobacillus gasseri* BC12	Ultracentrifugation (vesicles) Electrophoresis + LC-ESI-MS/MS (proteome)	15	Membrane ~ 44% Cytoplasm ~ 44% Secretory ~ 11%	- Foldase protein PrsA - Enolase 1 (Eno1) and 2 (Eno2) - Translation proteins: elongation factor (EF-Tu) and 30S ribosomal subunit (RpsD) - Metabolism proteins: AtpA, AtpD, AtpF, AtpH, Pyk, TpiA - Phosphonates transporter PhnC	[74]
*Lactobacillus crispatus* BC5	Ultracentrifugation (vesicles) Electrophoresis + LC-ESI-MS/MS (proteome)	11	Membrane ~ 45% Cytoplasm ~ 45% Secretory ~ 9%	- Enolase Eno1 - Translation proteins: 30S ribosomal subunit (RpsD) and 50S ribosomal subunits (RplB, RplU) - Metabolism proteins: AtpA, AtpD, AtpF - Phosphonates transporter PhnC	[74]
*Lactobacillus johnsonii* N6.2	Ultracentrifugation (vesicles) Electrophoresis + LC-MS/MS (proteome)	366	Cytoplasm + ribosomes 86% Secretory 14%	- Foldase protein PrsA - Aggregation promoting factors: Apf1, Apf2 - Translation proteins: 30S ribosome subunits (RpsB, RpsC, RpsE) and 50S ribosome subunit (RplA) - Metabolic proteins: PtsP, GalE, InuJ, RfbB, PfkA	[33]
*Bifidobacterium longum* NCC 2705	Ultracentrifugation (vesicles) Electrophoresis + LC-MS/MS (proteome)	24	Membrane 21% Cytoplasm 75%	- Iron transporter BL1134_04745 - Metabolism proteins: GltX, Pyk, GatA, GapA, SerS, Ppa - Translation proteins: elongation factor (EF-Tu), 30S ribosomal subunits (RpsC, RpsI), 50S ribosomal subunit (RplY)	[64]
*Propionibacterium freudenreichii* CIRM-BIA 129	SEC (vesicles) Electrophoresis + LC-ESI-MS/MS (proteome)	319	Membrane 16% Cytoplasm 75% Secretory 9%	- Enolase Eno1 - Aconitase Acn - Surface-layer proteins: SlpB, SplE, BopA, InlA - Antioxidative proteins: SodA, AhpC - Metabolic proteins: GlnA1, Gpi, Tpi1	[78]
	SEC (vesicles) Electrophoresis + LC-MS/MS (proteome)	391 (medium-dependent; 358 common for all)	Membrane 16.5% Cytoplasm 74% Secretory 9.5%	- Surface proteins: SlpB, SlpD, BopA, InlA - Metabolism proteins: LacZ, IolC, IolE1, AroH, NirA2 - Translation proteins: 30S ribosomal subunit (RpsG) and 50S ribosomal subunits (RplB, RplC, RplT, RplV) - Antioxidative protein AhpC	[79]
*Lactococcus lactis* FM-YL11	Ultracentrifugation (vesicles) Magnetic precipitation + LC-MS/MS (proteome)	1283	From the top 320 proteins: Membrane 16.5% Cytoplasm 74% Secretory 9.5%	- Translation proteins: elongation factor (EF-Tu), 30S ribosomal subunits (RpsA, RpsB, RpsC, RpsD, RpsE, RpsG, RpsK, RpsM), 50S ribosomal subunits (RplB, RplC, RplD, RplE, RplS, RplT, RplU) - Metabolic proteins: LacB, PyrG, LLT1_01140	[62]
*Pediococcus pentosaceus* **	Ultracentrifugation (vesicles) Electrophoresis + LC-MS/MS (proteome)	103	Membrane 9% Cytoplasm + ribosomes ~ 83% Secretory 5.5%	- Foldase protein PrsA - Enolase Eno1 - Translation proteins: elongation factors (EF-Tu, EF-G) and 50S ribosome subunits (RplD, RplO, RplQ) - Metabolic proteins: TpiA, ldhL	[42]

Abbreviations: *ND*, no data; DGC, density gradient centrifugation; LC-MS/MS, liquid chromatography with tandem mass spectrometry; nLC-MS/MS, nano-scale liquid chromatographic tandem mass spectrometry; LC-ESI-MS/MS, liquid chromatography electrospray ionization with tandem mass spectrometric; MALDI-TOF, matrix-assisted laser desorption/ionization-time of flight; SEC, size exclusion chromatography. * Total number of whole-length gene products, ** bacterial strain was not reported.

### 3.3. Biological Activities and Properties of EVs Produced by Probiotics

The last aspect considered in our review was the analysis of biological activities and properties of probiotics’ EVs. As reported in Table 2, a total amount of 54 articles were considered. In 23 of them, experiments were performed only in in vitro models, and 6 used only in vivo models, while the other 25 analyzed both (amongst these, 5 used ex vivo models). In our opinion, inclusion of both in vitro and in vivo models provides, undoubtedly, a better understanding of EVs’ activity. For example, as reported by Chen et al. [76], the use of murine models allows us to assess whether EVs could be transported to the femoral heads of glucocorticoid-treated mice after intragastric administration, thus providing information about EVs’ tissue distribution. We can assume that studying EVs’ properties addresses the same challenges of characterizing new probiotics. As described by Papadimitriou et al. [114], one of the most important advantage of in vitro assays is the ability to perform different screenings simultaneously, helping to evaluate potential interactions between probiotics and their products with the host. At the same time, these models are affected by some biases since the laboratory conditions only partially reproduce the in vivo situation. As well as probiotics needing to reach the desired body niches alive, EVs’ stability needs to be established since it depends on multiple factors. Thus, in vivo assays may be more appropriate because they can reproduce the complexity of the existing interactions. On the other hand, the main weakness of in vivo models is that they cannot be used for high throughput screening due to the increased cost and for ethical issues. For the above-mentioned reasons, a combination of in vitro and in vivo tests could represent an appropriate approach for EVs’ study [115].

Of the 54 articles considered, a large number of studies focused on the evaluation of EVs’ activity on the gastrointestinal system. From them, the most commonly applied models were Caco-2 cells, a model of the intestinal epithelial barrier, and C57BL/6 mice, one of the most adaptable animal models (Table 2). It is widely known that probiotics have a beneficial effect on the intestinal homeostasis, and this is obtained via multifactorial health-promoting activity [1,5,6]. In fact, several authors reported ability of probiotic EVs to enhance the intestinal barrier integrity by increasing the expression of tight junction (TJ) proteins, such as *ocldn*, *zo1*, *zo2,* and *zo3* [68], and by reducing *cldn-2* [67,69] (Table 2). Alterations of TJ barrier function and paracellular permeability are closely associated with the onset of metabolic diseases. All the previously-mentioned proteins aggregate into complexes located at the apical site of the lateral membranes of intestinal epithelial cells and regulate the selective passage of ions, solutes, and water. Occludins, the first identified integral membrane TJ proteins, create a barrier against macromolecules through the hemophilic interactions of their extracellular loops and so they have a crucial role in TJ structure and function [116]. ZO proteins are multi-domain proteins that provide an intracellular scaffold in TJs, creating a direct connection with the actin cytoskeleton and cytoskeleton-associated proteins; it is also recognized that ZO proteins have an important role in the regulation of TJ assembly [116]. Claudins, on the contrary to the two previously-described proteins, confer pore-like properties on TJs and regulate the selective passage of molecules in the paracellular pathways [116]. Of note is that increased claudin-2 expression by intestinal epithelial cells is correlated with colitis and inflammatory bowel disease [117]. As reported in Table 2, EVs produced by *A. muciniphila* can decrease the expression of *cldn-2*, thus regulating the integrity of the intestinal barrier and reducing inflammation [67]. Moreover, in the context of inflammatory bowel diseases (IBD), Hao et al. [93], Tong et al. [94], and Kang et al. [118] detected colitis amelioration in mice treated with EVs. In their studies, these authors used similar models, C57BL/6 and C57BL/6J mice with specific pathogen-free conditions, and colitis was induced by dextran sulfate sodium (DSS). DSS-treated mouse is the most widely used to obtain a good experimental model of ulcerative colitis (UC) since it leads to pathological alternations that are similar to what occurs in human UC [119]. In all three articles [93,94,118], EVs were administered by oral gavage in similar dosage and, despite the difference in the producer strain (*L. plantarum* Q7 [93], *L. rhamnosus* GG (ATCC 53103) [94], and *L. kefirgranum* PRCC-1301 [118]), the results obtained were comparable. They all reported a reversion of colon shortening and a downregulation of pro-inflammatory cytokines, including IL-1β, IL-2, IL-6, and TNF-α. It is worth noting that another common finding was the restoration of the gut microbiota homeostasis [82,93,94,118] (Table 2). Dysbiosis of intestinal microbiota, with reduction of probiotics and rise in pathogenic bacteria, represents a significant feature in UC patients. Consequently, the therapeutic potential of probiotic strains in UC has been examined by several researchers, who have identified different related mechanisms of action [120]. Thus, regarding the articles considered in this review, we can assume that EVs could be considered one of the bacterial products involved. In this context, Ma et al. [81] also highlighted the correlation between EVs treatment and mucus barrier integrity enhancement. This association was widely studied by Petersson et al. [121], who observed that the colonic mucus layer in germ-free mice was very thin compared with that observed in conventionally housed mice. Moreover, the administration of bacterial products (lipopolysaccharide and peptidoglycan) restored the normal mucus levels.

Five studies focused on the correlation between EVs and tumor development (Table 2). Using animal models, Tomasi et al. [122], Luo et al. [92], and Shi et al. [90] tested EVs of *E. coli* Nissle 1917, *A. muciniphila* ATCC BAA-835, and *L. paracasei* PC-H1, respectively. Melanoma, prostate cancer, and colorectal cancer were investigated, and comparable results were obtained despite different routes of administration and dosage; each author reported a reduction in tumor growth. Luo et al. [92] observed an upregulation of M1 macrophages and CD8^+^ lymphocytes expressing IFN-γ and GZMB, concluding that *A. muciniphila*-EVs stimulate anti-tumor immunity against prostate cancer. Shi et al. [90], instead, found out that the treatment with *L. paracasei*-EVs increased the expression level of Bax and decreased Bcl-2. These results were confirmed in both in vivo and in vitro models, and the authors confirmed that EVs can be taken up by colon cancer cells and inhibit their growth through apoptosis induction. As is well documented, many tumor types induce extensive systemic perturbations in the activity of the immune system, although the microbiome can modulate the systemic immunity and thus influence the outcome of tumor control strategies [123]. Fessler et al. [124] summarized the potential biological mechanisms of microbiome-mediated immune modulation: (1) bacterial translocation to different body districts may stimulate the immune response by providing microbial-derived, conserved antigens; (2) cross-reactive T cells primed against bacterial antigens might exert anti-tumor effects; (3) gut bacteria can release soluble immunomodulatory factors (IL-12, IFN-γ, and TNF-α) that then disseminate systematically and can activate dendritic cells. In this context, given the results obtained by the authors previously mentioned, it can be assumed that EVs could represent one of the effectors of these processes. For the research of Tomasi et al. [122] it is necessary to highlight that they tested *E. coli*-EVs engineered with a cancer-specific epitope and showed that the administration of these EVs, but not of wild type EVs, induced a reduction in tumor growth (Table 2). Given these results, we can assume that, unsurprisingly, not all probiotic strains may have the same health-promoting potential, although engineered EVs could represent a promising tool in cancer therapies. The remaining two articles [56,57] noted the same EVs’ properties on HepG2, SW480, and HT29 cells, highlighting an anti-proliferative effect on cancer cell lines (Table 2). According to Behzadi et al. [56], *L. rhamnosus*-EVs can increase the apoptotic index (*bax*/*bcl2* expression ratio) in liver cancer cells in a dose-dependent manner. Keyhani et al. [57], analyzing EVs of the same probiotic strain, reported an inhibitory effect on colon cancer cells too. Otherwise, in the latter article, a specific mechanism of action was not considered.

Other authors frequently reported that EVs have an immunomodulatory effect, which is related to their role in the regulation of different types of cytokines, chemokines, and antibodies [35,37,63,125] (Table 2). The influence of probiotics on the human immune system is not strictly related to pathological conditions, as previously discussed; a large amount of research, in fact, proved that the gut microbiota play a crucial role in the development and regulation of the host immune system and this complex interplay starts already during the birthing process. Under the stimulus of probiotic-derived products, intestinal epithelial cells may release thymic stromal lymphopoietin (TSLP), transforming growth factor-β (TGF-β), IL-25, and B cell activating factor (BAFF) [126]. Furthermore, T_H17_, T_Reg_, and IgA-producing cells development is also regulated by gut microbiota [127]. Different strains of probiotics can increase the number of dendritic cells and macrophages, and activate the latter through proinflammatory mediators, such as cytokines, reactive oxygen species or nuclear factor kB, and Toll-like receptor 2 pathways [128]. Fabrega et al. [63] determined that the presence of LPS in *E. coli*-EVs may explain the activation of IL-6, IL-8, and TNF-α, while the upregulation of IL-10 seems to be attributed to the presence of other vesicle factors (Table 2). At the same time, Morishita et al. [84,85] elucidated that EVs-mediated cytokine production is strictly related to their internalization. The release of TNF-α and IL-6 from cells treated with EVs was reduced in the presence of endocytosis and TLR2 inhibitors, with only one exception for RAW264.7 cells, for which no reduction in TNF-α was observed even after blocking clathrin-mediated endocytosis and micropinocytosis (Table 2). This suggests that several pathways could be involved in the EVs–cell interaction, and characterizing the main effectors is the key for the understanding of the immunomodulation in the host.

Interestingly, two articles considered the correlation between gut microbiota and the nervous system when focusing on EVs’ activity (Table 2). Choi et al. [129,130] found that EVs treatment reversed the expression of brain-derived neurotrophic factors (BDNFs) in HT22 cells and afforded antidepressant-like effects in C57BL/6 mice with stress-induced depression. In the first study, they examined whether *L. plantarum*-EVs treatment could block stress-induced, depressive-like behaviours in mice during the stress induction phase and during the post-stress phase. In both cases *L. plantarum*-EVs treatment restored the expression levels of BDNFs in the hippocampus and reduced depressive-like behaviors. This correlation was confirmed by their results obtained in vitro using HT22 cells. In the second study, they also considered EVs of *B. subtilis* and *A. muciniphila* and obtained comparable, although not identical, results. These data were also confirmed by several experiments that have proved the existence of the so-called ‘gut–brain axis’. Activation of proinflammatory cytokines, such as IL-1 and IL-6, has a certain association with the development of depression, so the impact of probiotics on immune homeostasis could help in the prevention or treatment of depression [128]. However, the detailed mechanisms of the action of EVs and their tissue distribution remain to be explored further.

Considering the influence of microbiota on the host health, two articles evaluated the effect of probiotics on another extraintestinal tissue—the skin [55,87] (Table 2). In this regard, Kim et al. [55] evaluated the therapeutic properties of *L. plantarum*-EVs on *S. aureus*-induced mouse atopic dermatitis model and on keratinocytes. The results showed that *L. plantarum*-EVs decrease skin inflammation by reducing the level of proinflammatory cytokines (IL-4 and IL-6). Since current treatment of atopic dermatitis involves the use of anti-inflammatory drugs and emollients, in order to compensate poor immune tolerance and barrier dysfunction, probiotics and their byproducts could represent an alternative option in the prevention or treatment of this disorder. Another article by Jo et al. [87] evaluated the effect of probiotic EVs on skin aging by using human dermal fibroblasts (CCD986sk) and a clinical trial among Korean women. As reported in Table 2, authors discovered that *L. plantarum*-EVs exert an anti-aging and anti-pigmentation effect and were able to positively regulate multiple pathways in fibroblasts.

**Figure 2:**
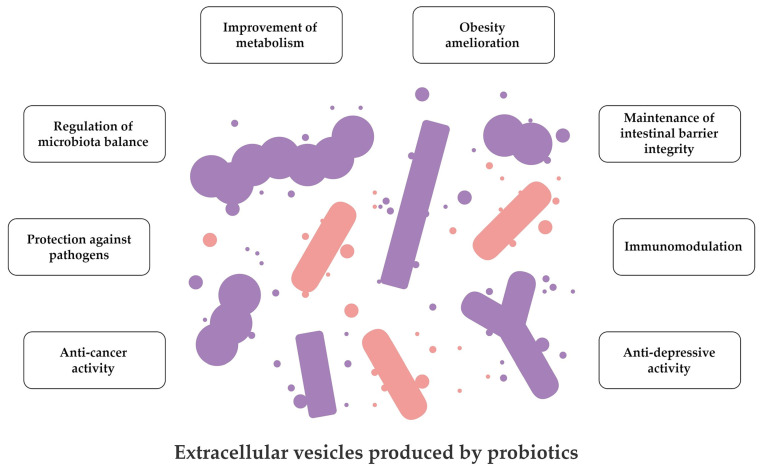
Graphical summary of data on the biological activities of extracellular vesicles secreted by probiotics.

Taken together, all the data summarised in Figure 2 and Table 2 suggest a huge variety of applications of probiotic EVs. Most of the studies confirmed their potential in protecting the intestinal barrier integrity and modulating host immune response in both physiological and disease-induced conditions. At the same time, there are many fields that still require further investigations on mechanisms by which probiotic EVs exert their activity. This knowledge could be then used to design innovative approaches in prevention and therapy of difficult-to-treat diseases.

**Table 2:** Biological activity of extracellular vesicles produced by probiotics reported in in vitro and in vivo models.

Bacterial Producer	In Vitro Model EVs Treatment (Duration)	In Vivo Model EVs Treatment (Duration)	Observations on EVs Activity	EVs Properties	Reference
*Escherichia coli* Nissle 1917	Caco-2 and T-84 cells (EPEC-infected) 0.1 mg/mL (24 h)	*ND*	- ↑ occludin and claudin-14	- Protection of intestinal barrier integrity against EPEC infection (enhancement of TJ)	[131]
	RAW264.7 murine macrophages1 µg/mL (16 h)	*ND*	- ↑ IL-4, IL-6, IL-12, and TNF-α - ↑ IL-10	- Anti-inflammatory properties - Enhanced immunomodulatory effect and antimicrobial function	[29]
OVA-*Escherichia coli* Nissle 1917, *Escherichia coli* BL21 Δ*ompA*	*ND*	Tumor in C57BL/6 and BALB/c female 4–8-week-old mice (administration: oral gavage) 10 µg (3–5 times)	- ↑ Tumor-specific T cells in the lamina propria - ↓ Tumor growth	- Protective activity against tumor development	[122]
*Escherichia coli* serotype O6:K5:H1	Caco-2 and HT-29 cells 10 mg/mL (8 h)	*ND*	- Activation of NOD-1 signaling and NF-κB - ↑ IL-6 and IL-8 - ↓ IκBα	- Maintenance of intestinal homeostasis	[132]
	Caco-2 and PMBCs cells 50 µg/mL (5–24 h)	Colon organ culture (ex vivo model) 50 µg/mL (5 h)	- ↑ IL-10, MIP1a, TNF-α, IL-6, and IL-8 by Caco-2/PBMCs co-culture - ↓ IL-12 and TGF-β in ex vivo model	- Modulation of the immune response - Anti-inflammatory properties	[63]
	Human Monocyte-Derived DCs 10 µg/mL (24 h)	*ND*	- ↑ 93 miRNAs and ↓ 64 miRNAs - ↑ miR-155, miR-let7i, and miR-146a - ↑ IFN-γ and IL-12	- Protection against pathogen infections - Anti-inflammatory/tolerogenic action	[133]
*Akkermansia muciniphila* ATCC BAA-835	Caco-2 cells 0.1, 0.5, and 5 μg (24 h)	*ND*	- ↑ *ocldn*, *zo1, zo3,* and *zo2* expression - ↓ *tlr4* and *trl2* expression	- Enhancement of intestinal barrier integrity - Anti-inflammatory properties	[68]
	Caco-2 cells 10 μg (24 h)	HFD induced and ND thirty male C57BL/6 mice (administration: oral gavage) 10 μg (5 weeks)	- ↑ *ZO-1, OCLDN*, and *CLDN-1* and ↓ *CLDN-2* expression in HDF mice - ↑ TLR-2 and ↓ TRL-4 expression in cells - ↓ blood glucose, cholesterol levels, and adipocyte dimensions in HDF mice - ↓ TNF-α, IL-6, and TLR-4 expression in HDF mice	- Obesity amelioration and prevention	[67]
*Akkermansia muciniphila* ATCC BAA-835	*ND*	NFD induced and NF 8-week-old male C57BL/6 mice (administration: oral gavage) 10 μg protein/200 μL (5 weeks)	- ↑ *tlr-2* and IL-10 - ↑ *zo-1* and *ocldn* and ↓ *cldn-2* - ↑ *angptl4* - ↓ *tlr-4, tnf-α,* and *tgf-β* - ↓ food intake and glucose level	- Preventive effect on obesity through enhancement of TJ	[69]
	Caco-2 cells (inflammation model) 0.1, 1, and 10 μg (4–8 h)	HFD in 6–8-week-old male C57BL/6 mice (administration: oral gavage) 10 μg (14 days)	- ↑ expression of occludin, zonal occludens, and claudin-5 in mice - ↑ glucose tolerance in mice - ↑ AMPK phosphorylation in cells - ↓ tight junction permeability in cells	- Improvement of gut permeability and metabolic functions (enhancement of tight junctions) - Anti-diabetes properties	[91]
	THP-1 and RAW264.7 10 μg/mL (24 h)	Prostate cancer RM-1 mice model (administration: injection) 40 μg per mouse (13 days)	- ↑ M1 macrophages in cancer in vitro - ↑ proportion of CD8+ and IFN-γ+ T cells in mice - ↓ 60% tumor growth in mice - ↓ proliferation of prostate cells	- Antitumor response and immunotherapy applications for prostate cancer	[92]
	LX-2 cells (inflammation model) 1, 10, 50 µg/mL (24 h)	Chronic liver injury in 7–8-week-old male C57BL/6 mice (administration: intraperitoneal injection) 50 µg protein/200 µL (4 weeks)	- ↓ TNF-α and IL-6 and ↑ IL-10 levels in mice - ↓ expression *of a-SMA, pdgf, timp*, and *Col1a1* genes in cells - ↓ *tlr-2* and *tlr-4* gene expression in cells	- Improvement of intestinal permeability - Modulation of inflammatory responses - Prevention of liver injury	[70]
	Caco-2 and Hep-G2 cells 50, 100 µg/mL (24 h)	*ND*	- ↑ mRNA level of FAAH and PPARα gene in both Caco-2 and Hep-G2 cells - ↑ mRNA level of PPARϒ gene in Caco-2 cells - ↑ mRNA level of the CB2R in Hep-G2 cells - ↑ transcription level of the PPARα gene in Hep-G2 cells - ↑ mRNA level of the PPARβ/δ gene in Hep-G2 cells - ↓ mRNA level of CB1R and CB2R in Caco-2 cells	- Prevention of metabolic disorders associated with obesity - Stimulation of fatty acid oxidation and energy metabolism - Control of the activity of ECS compartments (involved in obesity, metabolic disorders, and liver diseases)	[53]
*Akkermansia muciniphila* ATCC BAA-835	LX-2 cells (inflammation model) 1, 10, 50 µg/mL (24 h)	Livery injury in 8-week-old male C57BL/6 mice (administration: oral gavage) 50 µg protein/200 µL (4 weeks)	- ↑ mRNA level of *ppar-α, ppar-γ,* and *igf* in cells - ↓ *tlr-5* and *tlr-9* gene mRNA level in cells - ↓ TNF-α and IL-6 levels in mouse	- Enhancement of anti-inflammatory responses of the colon, adipose, and liver tissues	[134]
*A. muciniphila* ATCC BAA-835, *Faecalibacterium prausnitzii* A2-165c	Caco-2 cells 1 and 50 μg/mL (24 h)	*ND*	- ↑ serotonin level - ↑ expression of *Tph1, Htr3B, Htr2B, Slc6a4,* and *Htr4*	- Role in the homeostasis maintenance of the serotonin system	[15]
*A. muciniphila* ATCC BAA-835, *L. plantarum* KCTC 11401BP, *Bacillus subtilis* *	HT22 cells (stress model) 20 μg (24 h)	Chronic stress in 7-week-old male C57BL6 mice (administration: intraperitoneal injection) 6 μg/100 μL mouse per day (14 days)	- ↑ *Bdnf*, *Nt3*, and/or *Nt4/5* in mice - ↑ *Bdnf* and *Nt4/5* in cells - ↓ immobility in TST in mice	- Anti-depressive-like effect and restoration of stress levels (especially by *L. plantarum* EVs)	[130]
*Lactiplantibacillus plantarum* KCTC 11401BP	HT22 cells (stress model) 20 μg/mL (24 h)	Depression in 7-week-old male C57BL/6J mice (administration: intraperitoneal injection) 0.1, 0.18, and 0.27 μg/kg (1–35 days)	- ↑ *tBdnf*, *Bdnf1*, *Bdnf4*, and *Ngf* in cells - ↑ *Sirt1* in cells - ↑ *Bdnf1*, *Bdnf4*, and *Nt4/5* in mouse	- Antidepressant-like effects	[129]
	HaCaT cells and keratinocytes 0.1, 1, and 10 μg/mL (12 h)	*S. aureus* atopic dermatitis-induced mouse model (administration: oral gavage)	- ↓ IL-6 secretion in cells and mice stimulated with *S. aureus* EVs - ↓ epidermal thickening in mice	- Preventive effect on skin inflammation	[55]
*Lactiplantibacillus plantarum* APsulloc 331261	THP1 cells 10 μg/mL (48 h)	Human skin organ culture (ex vivo) 50 μg/mL (2–4 days)	- ↑ IL-10, IL-1β, and GM-CFS - ↑ M2-polarized cell markers	- Anti-inflammatory effect through macrophage polarization	[50]
*Lactiplantibacillus plantarum* WCFS1	Caco-2 cells 500 µL (24 h)	*C. elegans* Bristol N2 EVs isolated from 10^9^ CFU (1–15 days)	- ↑ *CTSB* and *REG3G* expression (cells) - ↑ gene expression of C-type lectin *clec-60* and the gut-specific cysteine protease *cpr-1* - (*C. elegans*)	- Antimicrobial effect - Host defense enhancement against infections	[32]
*Lactiplantibacillus plantarum* Q7	*ND*	Colitis in 4–5-week-old SPF male C57BL/6J mice(administration: gavage) 10/20 mg Q7-EVs group (0.5/1 mg/kg body weight) (1–18 days)	- ↑ *Bifidobacterium*, *Rikenellaceae*_RC9_gut_group, *Akkermansia*, Muribaculaceae, *Lactobacillus*, and *Alitipes* in gut microbiota - ↓ IL-1β, IL-2, IL-6, and TNF-α - ↓ colon shortening - ↓ spleen index	- Colitis alleviation - Regulation of intestinal microbiota - Anti-inflammatory properties	[93]
*Lactiplantibacillus plantarum* *	CCD-986Sk cells 0.625%, 1.25%, 5%, and 10% EVs (24 h)	Korean women in their 50s (administration: topically on the skin) (twice a day, 4 weeks)	- ↑ fibroblasts proliferation - ↑ expression of ECM components (Type 1 procollagen, filaggrin, HAS2) - ↑ water content in the skin - ↓ mRNA of MMP-1 - ↓ elastase activity - ↓ distribution and formation of wrinkles - ↓ pigmentation of the lesion sites	- Anti-aging and anti-pigmentation effect	[87]
*Lactiplantibacillus plantarum* YW11	Primary cortical neurons from C57BL/6 mice (OGD model) Co-culturing with EVs (24 h)	tMCAO (ischemic stroke model) in 10–12-week-old male C57BL/6 mice (administration: injection through the tail vein) 100 μg/day (3 days)	- ↑ miR-101a-3p expression and blocking of c-Fos/TGF-β axis in neurons - ↓ Bax and caspase 3 and ↑ Bcl-2 - ↓ neurological deficits and infarct size in tMCAO mice	- Anti-apoptotic effect on ischemic neurons both in vivo and in vitro	[86]
*Lacticaseibacillus casei* BL23	T84 and HT-29 cells 20 ng/mL to 10 μg/mL (24 h)	*ND*	- ↑ EGFR phosphorylation caused by P40 and P75 bounds to EVs	- Immunomodulatory effects	[35]
*Lacticaseibacillus casei* ATCC 393	Caco-2 cells 100 and 150 μg/mL (24 h)	*ND*	- ↑ IL-10, IL-4, IL-6, and GM-CSF - ↓ IFNγ - ↓ *TLR9* expression	- Immunomodulatory effects	[37]
*L. casei* DSMZ 20011, *L. plantarum* NCIMB 8826	Caco-2 and THP-1 cells (inflammation model) 5 × 10^11^–5 × 10^12^ EVs/mL (24 h)	*ND*	- ↑ IL-10 - ↓ TNF-α and IL-8	- Anti-inflammatory properties	[83]
	THP-1 cells (inflammation model) 1:2 EVs per well (48 h)	*ND*	- ↑ IL-10 - ↓ TNF-α	- Anti-inflammatory properties	[31]
*Lacticaseibacillus paracasei* *	RAW 264.7 cells (inflammation model) 0.1, 1, 10, 50 μg/mL (12 h) HT 29 cells (inflammation model) 500 ng/mL (12 h)	Acute colitis-induced 7-week-old male C57BL/6 mice (administration: oral gavage) 5 mg/day (12 days)	- ↑ ER-stress-associated proteins (CHOP, p-PERK, p-IRE1, and cleaved ATF6) in cells - ↑ IL-10 and TGFβ in both models - ↓ IL-1α, IL-1β, IL-2, TNFα, and NO in cells - ↓ COX-2 and iNOS expression in both models	- Anti-inflammatory effect through the activation of ER stress - Protective properties in an acute colitis-induced mouse model	[58]
*Lacticaseibacillus paracasei* PC-H1	Colorectal cancer cell line, HCT116, SW1116, and SW620 cells 200 μg/mL (24 h)	4-week-old female BALB/c nude mice (administration with HCT116 and EVs through subcutaneous injection) 200 μg/mL (30 days)	- ↑ apoptosis in cancer cells - ↓ growth of tumor tissue in mice	- Inhibition of colon cancer cell migration and invasion through apoptosis activation	[90]
*Lactobacillus crispatus* BC3, BC5; *Lactobacillus gasseri* BC12, BC13	Human T-lymphocyte MT-4 and Jurkat-tat cell lines 50 μL (1–72 h)	Human tissue cultures (ex vivo model) 10^8^ EVs/mL (12 days)	- ↓ HIV replication	- Anti-viral properties by EVs from *L. gasseri* B12	[74]
*Limosilactobacillus reuteri* BBC3	HD11 cells 10 µg/mL (6 h or 12 h) Splenic lymphocytes 10 μg/mL (12 h)	Broiler chicks (inflammation model) (administration: oral gavage) 200 μg/bird (21 days) Jejunum explant culture (ex vivo model) (inflammation model) 10 µg/mL (6 h)	- ↑ IL-10 and TGF-β (jejunum and cells) - ↓ TNF-α, IL-1β, IL-6, IL-17, IL-8, and MIP-1β in jejunum and cells	- Anti-inflammatory properties - Immunomodulatory effect	[34]
*Lacticaseibacillus rhamnosus* GG	HepG2 cells 50, 100, 150, and 200 μg (24 h)	*ND*	- ↑ *bac/bcl-2* gene expression - ↑ apoptosis	- Anti-proliferative effect on liver cancer cells	[56]
	SW480 and HT 29 cells (human colon cancer cell lines) 5–200 µg/mL (24 h)	*ND*	- ↑ expression of *cea* gene and CEA protein synthesis - inhibitory effects on both cell lines	- Anti-proliferative effect on cancer cells	[57]
	*ND*	Colitis-induced 4–5-week-old C57BL/6J male mice (administration: oral gavage) 1.2 mg/kg of body weight (14 days)	- ↑ the α-diversity of gut microbiota (Firmicutes and Bacteroidetes) - ↓ body weight - ↓ colon shortening - ↓ IL-1β, IL-2, IL-6, and TNF-α	- Anti-inflammatory effect - Colitis amelioration	[94]
*Lacticaseibacillus rhamnosus* JB-1	HT-29 and MODE-K cells 3 × 10^10^ EVs (2 h)	8- to 10-week-old SPF BALB/c male mice (administration: oral gavage) 3 × 10^10^ EVs (2 h)	- ↑ Toll-like receptor 2 (TLR2) - ↑ IL-10 - EVs contain immunologically active lipoteichoic acid (LTA)	- Enrollment of LTA on immunoregulatory activity	[88]
*L. rhamnosus* GG, *L. reuteri* DSM 17938	PBMCs cells 500:1, 100:1, and 20:1 (48 h)	*ND*	- ↑ IL-6, IL-10, IL-17A, and IFN-γ	- Immunomodulatory properties	[135]
*Lentilactobacillus kefirgranum* PRCC-1301	Caco-2 and HCT116 cells 0, 10, and 100 µg/mL (6–48 h)	Colitis-induced 6-week-old male C57BL/6 mice (administration: oral gavage) 3 mg/kg (3–14 days)	- ↑ ZO-1, claudin, and occludin in cells - ↓ IL-2, IL-8, and TNF-α in cells - ↓ shortening of the colon length in chronic colitis (mice)	- Enhancement of the intestinal barrier integrity (TJ) - Attenuation of chronic colitis - Anti-inflammatory effect	[118]
*Lentilactobacillus kefir* KCTC 3611, *Lentilactobacillus kefiranofaciens* KCTC 5075, *Lentilactobacillus kefirgranum* KCTC 5086	Caco-2 cells (inflammation model) 1 × 10^9^ EVs/mL (24 h)	IBD-induced 8-week-old male BALB mice (administration: oral gavage) (3 × 10^8^ or 3 × 10^10^ EVs/head)	- ↓ IL-8 in cells - ↓ TNBS-induced infiltration of transmural leukocyte and loss of goblet cells in mice	- Preventing of enterorrhagia and diarrhea - Reduction of MPO activity - Anti-inflammatory properties	[60]
*Latilactobacillus sakei* NBRC 15893	PP and BMDCs from BALB/c mice (female, 7–14 weeks old) 30 μg protein/mL (4 days)	*ND*	- ↑ IgA in PP cells - ↑ expression *of IL-6, IL-10, IL-12, TNF-α,* and *NOS2* in BMDCs - ↑ NO and RA production	- Immunomodulatory effect	[125]
	PP cells 37 μg/mL EVs (1–4 days)	*ND*	- ↑ IL-6 production via TLR2 signal - ↑ IgA level	- Activation of the mucosal immune system	[61]
*Ligilactobacillus animalis* ATCC 35046	HMECs, MLO-Y4, MC3T3-E1, and BMSCs(MPS-treated) 10 μg/mL (6–24 h)	GC-induced ONFH male C57BL/6J mice (administration: oral gavage) 30 μg/200 μL (once a week)	- ↑ tube formation of the MPS-treated HMECs - ↑ BMSCs mineralization in BMSCs - ↑ blood vessel volume and numbers of CD31-positive endothelial cells and OCN-stained osteoblasts in MPS-treated mice - ↓ MPS-induced negative effect on osteogenic differentiation of BMSCs - ↓ MPS-induced apoptosis of HMECs, MLO-Y4, MC3T3-E1, and BMSCs - ↓ apoptotic cell number and serum levels of IL-2 and IFN-γ in MPS-treated mice	- Prevention of GC-induced ONFH	[76]
*Lactobacillus johnsonii* N6.2	Pancreatic cell line βlox5, Caco-2, Jurkat, and THP-1 cells 10^8^ or 10^10^ EVs/mL (2–8 h)	Pancreatic islets isolated from human donors(ex vivo model) 6 × 10^9^ EVs/mL (5 h)	- ↑ OAS1, OAS3, AHR pathways, CYP1A1, and CYP1B1 in βlox5 cells - ↑ GLUT6, SREBF1, PRKACA, and mRNA of GLP1R - ↑ MTA2 and STC2 and ↓ SOD1 in pancreatic islets - ↑ CYP1A1 in THP-1 and Caco-2 cells - ↑ TNFα, IL-1β, IL10, TLR2, and TLR7 in THP-1 cells - ↑ T-STAT3/actin and pYSTAT3/T-STAT3 ratio in THP-1 cells	- Dose-dependent protection against apoptosis in pancreatic beta cell line - Stimulation of insulin release in human islets under high glucose stimulation - Regulation of intra-islets environment - Potential protective effect against insulin - resistance and in type 2 diabetes metabolic syndrome	[136]
*Lactococcus lactis* *	Dendritic cells isolated from asthmatic patients 10 μg/mL (24 h)	Allergic asthma-induced 6-week-old female BALB/c mice (administration: intranasally) 10 μg/20 μL PBS (5 days)	- ↑ IFN-γ in the BALF of mice - ↑ secretion of IL-12p70 from dendritic cells - ↓ IL-5 and IL-13 - ↓ expression of GATA-3 and phosphorylation of STAT6	- Immuno-modulating effect in allergic airway inflammation - Regulation of allergic response by enhancing Th1 immune activation	[95]
*Bifidobacterium longum* KACC 91563	PP, T cells, B cells, eosinophils, and BMCCs from mice 2 µg/mL (2 h)	Food allergy-induced 6- to 8-week-old BALB/c mice (administration: oral gavage) EVs from 10^9^ CFU/mouse (2 weeks)	- ↑ annexin V+ apoptotic cells in mast cells - ↓ mast cell numbers	- Therapeutic effect on food allergy through apoptosis	[137]
*Bifidobacterium bifidum* LMG 13195	Monocyte-derived DCs and naïve T cells 0.1 μg/mL (48 h)	*ND*	- ↑ IL-10 - ↑ differentiation of CD25^high^ FOXP3^high^ CD127^−/low^ Treg cells	- Immunotherapy application (SIT vaccines)	[138]
*B. longum* *, *L. plantarum* WCFS1	DC2.4 and RAW264.7 cells 0.01 or 0.1 μg/well (6–24 h)	*ND*	- ↑ TNF-α and IL-6 - ↓ TNF-α and IL-6 from DC2.4 after inhibition of the clathrin-mediated endocytosis or macropinocytosis pathway	- Immunomodulatory effect through clathrin-mediated endocytosis and macropinocytosis	[85]
	DC2.4 and RAW264.7 cells 0.5 μg/well (6 h)	*ND*	- ↑ TNF-α and IL-6 - ↓ TNF-α and IL-6 in the presence of TLR2 inhibition	- Immunomodulatory effect through TLR-2 signaling	[84]
*Propionibacterium freudenreichii* CIRM-BIA 129	HT-29 cells (inflammation model) 10^9^ EVs/mL (1 h)	*ND*	- ↓ NF-kB activation - ↓ IL-8	- Anti-inflammatory and immunomodulatory properties through NF-kB pathway	[78]
	HT-29 cells (inflammation model) 10^9^ EVs/mL (24 h)	*ND*	- ↓ IL-8 especially with LPS-induced inflammation - ↓ NF-kB activation especially with LPS-induced inflammation	- Anti-inflammatory properties (depending on the media conditions)	[79]
*Bacillus subtilis* 168	Caco-2 cells 1.3 × 10^9^ EVs (0–4 h)	*ND*	- - Uptake of MVs by transcytosis (dose-dependent)	- Immunomodulatory properties through transcytosis	[80]
*Clostridium butyricum* MIYAIRI 588	*ND*	Ulcerative colitis in 40–60-day-old male C57BL/6 mice (administration: intragastrically) 15 μg/200 μL (once a day, 5 days)	- Inhibition of disease progression and reduction of mortality rate - ↑ MUC2 and ZO-1 - ↑ Bacilli, Bacteroidia, and Verrucomicrobiae - ↑ proportion of M2 cells and ↓ M1 cells in the gut - ↓ inflammation and tissue damage - ↓ Enterobacteriaceae, Helicobacteraceae, and Lachnospiraceae	- Re-establishment of M1/M2 in UC models - Reversing of the gut microbial dysbiosis - Protective effect against UC	[82]
	*ND*	Ulcerative colitis-induced male C57BL6J mice (administration: oral gavage) 50 μg/day (11 days)	- ↑ expression of *Muc1*, *Muc2*, *Muc3*, *Muc4*, *Claudin1*, *Claudin3*, *Zo-1*, *Svs1*, *Doxl2*, and *Rik* genes - ↑ ERK1 and ERK2 cascades - ↑ *Ruminiclostridium* and Ruminococcaceae in the gut - ↓ LPS, IL-6, and TNF-α - ↓ inflammatory cell infiltration and mucus layer damage in the colon - ↓ expression of *Tlr4*, *Nf-kb*, *TNF-𝛼*, *F4/80*, *Cd11c*, *Mcp1*, and *Ccl5* genes	- Protection of gut barrier function - Modulation of gut microbiota homeostasis - Reduction of ulcerative colitis symptoms - Anti-inflammatory properties	[81]
*Leuconostoc holzapfelii* GFC1203H, *L. plantarum* *, *B. longum* *, *B. animalis* *, *L. acidophilus* *	Human HFDP cells 1, 2.5, 5, and 10 μg/mL (6 h, 12 h, 24 h)	*ND*	- ↑ cell migration and cell proliferation (7 to 24%) - ↑ Bcl-2 and Bax - ↑ Wnt5A, Wnt10B, β-catenin, VSC, Lef1, BAMBI, and BMP-2 - ↓ sub-G1 phase and ↑ G2/M phase - ↓ Caspase-3 activity	- Anti-apoptotic effect - Induction of cell division, migration, and proliferation - Induction of hair growth	[77]
*Pediococcus pentosaceus* *, *Ligilactobacillus salivarius* *	E.G7–EL4 and HEK-BLUE hTLR2, BMDCsMouse splenocytes (inflammation model) 0.2, 1, and 5 mg/mL (24 h)	Liver-fibrosis in 6- to 8-week-old male C57BL/6 mice (administration: injection) 10 μg/mouse (14 days)	- ↓ aSMA expression - ↑ collagen in the liver - ↑ M2 polarization - ↑ TLR-2 signaling	- Immunomodulatory effect and inflammatory properties	[42]

Abbreviations: *ND*, no data; MVs, membrane vesicles; EPEC, enteropathogenic *Escherichia coli*; TJ, tight junctions; TNF, tumor necrosis factor; IL, interleukin; IκBα, nuclear factor of kappa light polypeptide gene enhancer in B-cells inhibitor; NOD, nucleotide-binding oligomerization domain; TGF, transforming growth factor; TLR. toll-like receptor; HFD, high-fat diet; ND, normal diet; PP, Peyer’s Patch; IgA, immunoglobulin A; ZO, zonula occludens; OCLDN, occludin; CLDN, claudin; TLR, toll-like receptor; PPAR, peroxisome proliferator-activated receptors; Angptl4, angiopoietin-like 4; HT-29, human colonic epithelial; MODE-K, mouse duodenal epithelial; GC, glucocorticoid; TST, tail suspension test; BDNF, brain-derived neurotrophic factor; Sirt1, sirtuin 1; GM-CFS, granulocyte-macrophage colony-stimulating factor; *CTSB*, cysteine proteinase; *REG3G*, C-type Lectin; SIT, allergen-specific immunotherapy; LTA, lipoteichoic acid; NFKβ, nuclear factor kappa-light-chain-enhancer of activated B cells; LPS, lipopolysaccharides; CG, glucocorticoid; Sirt1, Sirtuin 1; PMBCs, peripheral blood mononuclear cells; ER, endoplasmatic reticulum; BMCCs, bone marrow-derived mast cells; MPO, myeloperoxidase; HMECs, human microvascular endothelial cells; MLO-Y4, mouse long bone osteocyte-Y4; MC3T3-E1, mouse preosteoblast cells; BMSCs, mouse bone marrow mesenchymal stem cells; ONFH, osteonecrosis of the femoral head; MPS, methylprednisolone; CB1R and CB2R, cannabinoid receptors; FAAH, fatty acid amide hydrolase; PPARs, peroxisome proliferator-activated receptors; ECS, endocannabinoid system; CCD-986Sk, human dermal fibroblasts; MMP-1, matrix metalloproteinase-1; ECM, extracellular matrix; HAS2, hyaluronidase 2; CEA, carcinoembryonic antigen; OVA, ovalbumin; BALF, bronchoalveolar lavage fluid; UC, ulcerative colitis; MUC2, mucin 2; ZO-1, zonula occludens protein 1; M2, M2 macrophages; M1, M1 macrophages; OAS, 2′,5′-oligoadenylate synthetase; AHR, aryl hydrocarbon receptor; CYP1A1 and CYP1B1, cytochrome P450 superfamily enzymes; GLUT6, glucose transporter 6; SREBF1, sterol regulatory element binding transcription factor 1; GLP1R, glucagon-like peptide 1 receptor; PRKACA, protein kinase catalytic subunit α; MTA2, metastasis-associated 1 family member 2; STC2, stanniocalcin-2; SOD1, superoxide dismutase 1; tMCAO, transient middle cerebral artery occlusion; OGD, oxygen-glucose deprivation; HFDPCs, human hair follicle dermal papilla cells. * Bacterial strain was not reported.

### 3.4. Future Application and Perspectives on EVs Produced by Probiotics

Unquestionably, we consider it very optimistic to find an exponential increase over the last decade in the number of publications on the production of probiotic EVs. For example, in 2012, only a single research article was published, while, in the first half of 2022 alone, as many as 15 original papers were released. Because of such a high dynamic of new articles appearing, during our analysis of the already collected literature and preparation of the central core of the manuscript’s discussion, we were unable to include newly-published articles from the second half of 2022. In this regard, however, we would like to point out that seven more original articles appeared during this time. These studies showed the protective effect of probiotic EVs on atopic dermatitis [139] and various intestinal pathologies (chemoresistant colorectal cancer [140] or intestinal infections made by enterotoxigenic *E. coli* [141]). It was also noticed that probiotic EVs have a strong immunomodulatory effect on the human body (including the intestines) [142], and that they can be used in the design of innovative vaccines against infectious diseases [143] and cancers [122]. In the last publication, attention was drawn to the participation of prophages in the secretion of EVs by the tested probiotic bacteria [144].

The latest research, together with the papers already analyzed in this review, lead us to highlight multiple applications of probiotic EVs. As previously mentioned, the close interconnection between probiotics and the gastrointestinal system is of great interest. In this context, EVs could represent a new strategy for the treatment of metabolic diseases, such as diabetes and obesity. There is evidence supporting the role of diet in shaping the host microbiota and the release of gut microbiota EVs, which, in turn, can exert their beneficial effect on human gut homeostasis [145]. In addition, another study showed that probiotic-derived EVs have a protective effect on Caco-2/HT29-MTX co-cultures exposed to enterotoxigenic *E. coli*, confirming the role of this bacterial structure in the maintenance of intestinal barrier integrity [141]. Considering their application in perspective, some authors also suggest that the administration of probiotic EVs alone could be a safer alternative than delivering live probiotics, especially in immunocompromised patients [146,147,148]. Moreover, in relation to some probiotic strains, the use of EVs alone could also represent an advantage in terms of efficacy. For example, Pang et al. [141] noted that EVs of *L. reuteri* DSM 17938 are more effective in the treatment of infantile colic compared to the bacterial cells of this strain.

Taking into account their biological properties, probiotic EVs could also be used for the treatment of certain neurological diseases. In fact, having the potential to cross the blood–brain barrier, they could represent efficient transporters for the delivery of drugs into the central nervous system [11]. Yuan et al. [149] and Sun et al. [150] extensively reported in their reviews the potential application of EVs-based strategies in the treatment of neurological disorders. Although in those papers only eukaryotic cells-based vesicles were considered, we cannot ignore the fact that they show comparable structure and properties to bacterial ones, which lead us to believe that a similar application of probiotics-derived EVs is reasonable. In a recent work, González-Lozano et al. [148] reported several examples of EVs’ applications in neurological disorders, focusing on their ability to transport active bacterial-produced compounds through different body districts. In addition, it is worth highlighting that the possibility to engineer EVs to improve their properties also exists. In this context, the surface modification can improve the EVs’ targeting capability and, as a consequence, their therapeutic potential [150]. To sum up, EVs biocompatibility, size, and drug delivering capabilities make them promising tools for future biomedical applications [147].

### 3.5. Challenges and Limitations of Articles Focusing on EVs Produced by Probiotics

As a summary, below, we would like to draw attention to a few challenges and limitations, the consideration of which may help the scientific community in an even more robust and reliable study of EVs produced by probiotics in the future.

We discovered that most research on the above topic focuses on the properties of EVs produced by Gram-positive bacteria; however, research on *E. coli* or *A. muciniphila* shows that the health-promoting properties of EVs of Gram-negative bacteria can be equally valuable (Table S1 and Table 2). It is also worth mentioning that other probiotic microorganisms, including yeasts of the genus *Saccharomyces*, have many health-benefiting properties [151] and EVs produced by them could possibly constitute very valuable therapeutics. For this reason, we would like to encourage scientists to increase the pool of tested microorganisms not only with new strains of the bacterial species tested so far, but also with novel genera or families of microorganisms [152,153].

The second aspect we would like to discuss is insufficiently low attention paid to characterizing biological and physicochemical properties of EVs produced by probiotics. As we proved in Figure 1 and Table S1, when undertaking such an analysis, it is mostly limited only to estimating the size of EVs. Other parameters were determined either rarely (electric charge and quantity) or extremely rarely (spatial orientation of EVs’ membranes or biological origin). Since we have already discussed the subject of electric charge and quantity of probiotic EVs, here, we would like to pay special attention to the second group of parameters. In none of the articles we analyzed, the spatial orientation of EVs (inside-out or right-side-out) was determined. The issue of the biological origin of EVs, and, thus, the precise nomenclature of isolated structures, has also been addressed sporadically. In most of the original articles, the term ‘extracellular vesicles’ (43) or ‘membrane vesicles’ (16) was used, while the presence of EVs’ subpopulations was included in only one article [51]. It is worth mentioning that cell lysis can be a significant source of EVs, and, hence, determining its intensity is also of importance [96,112]. Again, however, this phenomenon was rarely established (only in two publications [62,66]). In connection to the above description, we would like to sensitize scientists to increase their attention toward characterizing the properties of probiotic EVs and include “minimal information for studies of extracellular vesicles”, as recommended by the International Society for Extracellular Vesicles [18].

Another aspect we would like to highlight is the proteomic analysis of probiotic EVs. We noticed that, despite the relatively high methodological homogeneity, consisting of ultracentrifugation followed by electrophoresis combined with LC-MS/MS or MALDI-TOF, a large discrepancy within the data was noticed (Table 1). The total number of identified proteins (understood as whole-length gene products) ranged from as low as a dozen [74] to as high as over a thousand [54,62]. Still, however, in most articles, these values were within the range of several hundred. The phenomenon of under- or over-representation of identified EVs’ proteins may be caused by two sources—the sensitivity of the research techniques and the level of contamination of the EVs’ proteome with proteins derived from bacterial cells secreting these structures [40,96,97]. In line with this, we propose to make serious considerations for changing the techniques of isolation and analysis of EVs if the proteome of these structures is either too sparse or too numerous (especially when over-represented by cytoplasmic proteins).

Finally, the last issue worth recalling is the scope of the conducted research. In Table 2, we extensively presented the data of original articles describing biological activities of EVs produced by probiotics. As it can be relatively easily observed, a large amount of research on EVs produced by probiotics focuses on the evaluation of the biological effect of these structures only on the gastrointestinal system. For obvious reasons, including the ingestible administration of probiotic EVs, the action of these structures on the digestive system is highly intuitive and justified [154,155]. However, it should still be remembered that EVs, due to their nanometric dimensions, can reach various tissues of the host, and, therefore, their effect on different types of human cells should be discovered [13,156]. As described in this review, a good example of a different approach to evaluating the biological activity of probiotic EVs is the original article aimed at the skin [55,87] or the nervous system [129,130]. Undoubtedly, the health-promoting impact of probiotic EVs on the host should be extended to many other organs untested yet, such as the oral cavity, cardiovascular system, respiratory system, or genitourinary system.

## 4. Conclusions

Many decades of numerous studies on probiotics have confirmed their health-promoting effect on humans. Despite this, knowledge about the activity of EVs produced by probiotic microorganisms is still in its infancy. As our detailed review of the literature shows, in the last decade, the awareness on the usefulness of these structures is, however, dynamically growing. A broad variety of benefits of using EVs secreted by probiotics have already been shown, including regulation of intestinal homeostasis on both microbiota and host metabolism levels, anti-depressive activity, and immunostimulation, leading to a better control of microbial and carcinogenic disorders. We hope that the coming years will bring even more groundbreaking discoveries on these topics.

## Supplementary Materials

The following supporting information can be downloaded at: https://www.mdpi.com/article/10.3390/pharmaceutics15020522/s1, Table S1: Detailed data on physicochemical properties of extracellular vesicles produced by probiotics.
