# Supplementary material for: Extracellular Vesicles of Probiotics: Shedding Light on the Biological Activity and Future Applications
Source: Pharmaceutics. 2023 Feb 4;15(2):522. doi: 10.3390/pharmaceutics15020522 (PMC9967243; doi:10.3390/pharmaceutics15020522)
Supplement: Supplementary file 1 [file pharmaceutics-15-00522-s001.zip › pharmaceutics-2146722-supplementary.pdf]

Table S1. Detailed data on physicochemical properties of extracellular vesicles produced by probiotics.

Bacterial producer	Properties of membrane vesicles			Reference
	Size *	Charge	Amount	
**Gram-negative bacteria**				
Escherichia coli Nissle 1917	20 – 60 nm (TEM)	*ND*	*ND*	[1–3]
	20 – 60 nm (TEM)	*ND*	$1.16 \times 10^{-3} \pm 3.7 \times 10^{-4}$ relative fluorescence units/colony forming units (spectrophotometry)	[4]
	28.2 ± 9.54 nm (TEM)	*ND*	*ND*	[5]
	50 – 150 nm (TEM + SEM)<br>~99 nm (NTA)	*ND*	*ND*	[6]
	149 – 189 nm (NTA)	*ND*	$7.8 \times 10^{10} – 1.9 \times 10^{12}$ particles/mL (extraction method-dependent; NTA)	[7]
Akkermansia muciniphila ATCC BAA-835	40 – 150 nm (SEM)	*ND*	*ND*	[8–11]
	40 – 60 nm (DLS)	*ND*	*ND*	[12]
	181.9 ± 42.4 nm (DLS)	*ND*	*ND*	[13]
	20 – 200 nm (TEM)	*ND*	*ND*	[14]
	30 – 300 nm (TEM)	*ND*	*ND*	[15]
**Gram-positive bacteria**				
Lactiplantibacillus plantarum BGAN8	20 – 140 nm (cryo-TEM)	*ND*	*ND*	[16]
Lactiplantibacillus plantarum Q7	70 – 500 nm (DLS)	*ND*	*ND*	[17]
Lactiplantibacillus plantarum KCTC 11401BP	20 – 100 nm (TEM)	*ND*	*ND*	[18]
Lactiplantibacillus plantarum BCRC 10357	~124 – 130 nm (NTA)	*ND*	$3 – 3.8 \times 10^{8}$ particles/mL (NTA)	[19]
Lactiplantibacillus plantarum P8	100 – 150 nm (TEM)<br>~125 nm (NTA)	*ND*	*ND*	[20]
Lactiplantibacillus plantarum NCIMB 8826	50 – 200 nm (cryo-TEM)<br>116 ± 9 nm (NTA)	−3.3 ± 0.2 mV to −5.6 ± 2.0 mV (DLS)	$7.2 \times 10^{10} – 1.7 \times 10^{12}$ particles/mL (condition-dependent; NTA)	[21]
	117 ± 24 nm (NTA)	*ND*	6×10^{11} particles/mL (NTA)	[22]

Table S1. *Cont.*

Bacterial producer	Properties of membrane vesicles			Reference
	Size *	Charge	Amount	
Lactiplantibacillus plantarum WCFS1	30 – 200 nm (TEM)<br>31 – 200 nm (NTA)	*ND*	*ND*	[23]
	108 ± 0.8 nm (NTA)	−27.2 ± 2.03 mV (NTA)	*ND*	[24]
	127 nm (NTA)	~ −10 mV (NTA)	*ND*	[25]
Lactiplantibacillus plantarum APsulloc 331261	72 – 84 nm (DLS)	*ND*	2.95 – 5.75 × 10^{10} particles/mL (TRPS)	[26]
	104 ± 42.4 nm (ultracentrifugation-purified; DLS)<br>83 ± 20.3 nm (density-purified; DLS)	*ND*	2.45 – 3.06 × 10^{10} particles/mL (both extraction methods; TRPS)	[27]
Lactiplantibacillus plantarum YW11	66.96 ± 5.83 nm (NTA)	*ND*	*ND*	[28]
Lactiplantibacillus plantarum **	126.5 ± 56.4 nm (NTA)	*ND*	9.1 × 10^9 particles/mL (NTA)	[29]
Lacticaseibacillus casei BL23	13 – 28 nm (TEM)<br>~24 nm (DLS)	*ND*	*ND*	[30]
	47 ± 3 nm (DLS)<br>48 ± 3 nm (SEM)<br>33 ± 3 nm (AFM)	−8.7 ± 1.9 mV (DLS)	*ND*	[31]
Lacticaseibacillus casei ATCC 393	10 – 300 nm (AFM)<br>143 ± 52 nm (NTA)<br>~70 nm and ~250 nm (DLS)	~ −17 mV (DLS)	3 × 10^9 to 1 × 10^{10} particles/mL (NTA)	[32]
	140.7 ± 20.3 nm (SEM)<br>~ 227.3 nm (DLS)	−0.5 mV (DLS)	*ND*	[33]
Lacticaseibacillus casei DSMZ 20011	50 – 200 nm (cryo-TEM)<br>116 ± 5 nm (NTA)	−6.3 ± 0.7 mV to ~ −13 mV (DLS)	3.3 × 10^{11} – 4.4 × 10^{12} particles/mL (condition-dependent; NTA)	[21]
	113 ± 12 nm (NTA)	*ND*	2 × 10^{12} particles/mL (NTA)	[22]
Lacticaseibacillus rhamnosus GG	30 – 100 nm (TEM)	*ND*	*ND*	[34]
	161.9 ± 54.8 nm (DLS)	*ND*	*ND*	[35]
	50 – 150 nm (TEM)	*ND*	*ND*	[36]

Table S1. *Cont.*

Bacterial producer	Properties of membrane vesicles			Reference
	Size *	Charge	Amount	
Lacticaseibacillus rhamnosus JB-1	~130 nm (NTA)	*ND*	3.1 × 10^{11} particles/mL (NTA)	[37]
	~130 nm (NTA)	*ND*	2.3 – 2.6 × 10^{10} particles/mL (NTA)	[38]
Lacticaseibacillus paracasei PC-H1	~ 200 nm (NTA)	*ND*	*ND*	[39]
Lacticaseibacillus paracasei **	20 – 100 nm (TEM)	*ND*	*ND*	[40]
Lactobacillus gasseri JCM 1131^T	~ 130 nm (TEM)	*ND*	*ND*	[41]
Lactobacillus gasseri BC12 and BC13	~ 135 nm (NTA)	*ND*	5.87 × 10^{10} to 1.32 × 10^{11} particles/mL (NTA)	[42]
Lactobacillus acidophilus ATCC 53544	10 – 300 nm (AFM)<br>~142 ± 64 nm (NTA)<br>~35 nm and ~200 nm (DLS)	~ −11 mV (DLS)	3 × 10^9 to 1 × 10^{10} particles/mL (NTA)	[32]
Lactobacillus johnsonii N6.2	95.8 ± 46.5 nm (TEM)<br>99.2 ± 48.3 nm (cryo-TEM)<br>96.3 ± 58.6 (SEM)<br>~124 nm (NTA)	*ND*	1.45 x 10^9 particles/mL (NTA)	[43]
Lactobacillus crispatus BC3 and BC5	~ 130 – 140 nm (NTA)	*ND*	1.18 – 3.26 × 10^{10} particles/mL (NTA)	[42]
Limosilactobacillus reuteri DSM 17938	50 – 150 nm (TEM)<br>~210 – 240 nm (DLS)<br>~210 – 240 nm (NTA)	−13.4 mV for biofilm vesicles (DLS)<br>−39.8 mV for planktonic vesicles (DLS)	1.1 – 2.0 × 10^9 particles/mL (NTA)	[44]
	130.7 – 388.11 nm (planktonic vesicles; DLS)<br>2753 nm (biofilm vesicles; DLS)	−36.6 ± 0.4 mV (planktonic vesicles; DLS)<br>−24.6 ± 2.2 mV (biofilm vesicles; DLS)	3.38 × 10^7 planktonic vesicles/mL (flow cytometry)<br>4.48 × 10^7 biofilm vesicles/mL (flow cytometry)	[45]
	156.3 ± 2.1 nm (NTA)	*ND*	*ND*	[46]
Limosilactobacillus reuteri ATCC 23272	10 – 300 nm (AFM)<br>143 ± 55 nm (NTA)<br>~150 nm (DLS)	~ −8 mV (DLS)	3 × 10^9 to 1 × 10^{10} particles/mL (NTA)	[32]
Limosilactobacillus reuteri BBC3	50 – 150 nm (TEM + SEM)<br>60 – 250 nm (NTA)	*ND*	*ND*	[47]

Table S1. *Cont.*

Bacterial producer	Properties of membrane vesicles			Reference
	Size *	Charge	Amount	
Lentilactobacillus kefir KCTC 3611 *Lentilactobacillus kefiranofaciens* KCTC 5075 *Lentilactobacillus kefirgranum* KCTC 5086	80 – 400 nm (cryo-TEM)	*ND*	*ND*	[48]
Latilactobacillus sakei NBRC15893	30 – 400 nm (TEM)	*ND*	*ND*	[49]
Ligilactobacillus animalis ATCC 35046	118.8 ± 49.5 nm (NTA)	*ND*	3.7 – 4.6 × 10^{10} particles/mL (NTA)	[50]
Leuconostoc holzapfelii GFC1203H	~104.6 nm (NTA)	*ND*	2.03 × 10^{11} particles/mL (NTA)	[51]
Pediococcus pentosaceus **	~325 – 425 nm (DLS)	–35 to –45 mV (DLS)	*ND*	[52]
Lactococcus lactis FM-YL11	50 – 300 nm (TEM)	*ND*	*ND*	[53]
Lactococcus lactis **	60 – 100 nm (DLS)	*ND*	*ND*	[54]
Bifidobacterium longum NCC2705	50 – 80 nm (TEM)	*ND*	*ND*	[55]
Bifidobacterium longum **	126 ± 3.78 nm (NTA)	−10.8 ± 1.53 mV (NTA)	*ND*	[24]
	109 nm (NTA)	~ −8 mV (NTA)	*ND*	[25]
Propionibacterium freudenreichii CIRM-BIA 129	~85 nm (NTA)	*ND*	*ND*	[56]
	100.9 ± 3.2 nm or 117.9 ± 7.9 nm (medium-dependent; NTA)	*ND*	9.4 × 10^{11} to 2.2 × 10^{12} particles/mL (medium-dependent; NTA)	[57]
Bacillus subtilis 168	142 ± 14 nm (DLS)<br>52 ± 3 nm (SEM)<br>310 ± 5 nm (AFM)	−18.2 ± 1.7 mV (DLS)	*ND*	[31]
	115 ± 27 nm (NTA)	*ND*	*ND*	[58]
Faecalibacterium prausnitzii A2-165	20 – 200 nm (TEM)	*ND*	*ND*	[14]

Table S1. *Cont.*

Bacterial producer	Properties of membrane vesicles			Reference
	Size *	Charge	Amount	
Clostridium butyricum **	152 nm (NTA)	~ −10 mV (NTA)	*ND*	[25]
Clostridium butyricum MIYAIRI II 588	149.3 ± 50.9 nm (NTA)	*ND*	*ND*	[59]
	30 – 200 nm (NTA)	*ND*	*ND*	[60]

Abbreviations: ND, no data; TEM, Transmission electron microscopy; cryo-TEM, Cryogenic transmission electron microscopy; SEM, Scanning electron microscope; AFM, Atomic force microscopy; DLS, Dynamic light scattering; NTA, Nanoparticle tracking analysis; TRSP, Tunable resistive pulse sensing. * In case of the data determining the size of membrane vesicles using DLS or NTA the reported values constitute the highest reading peak. ** Bacterial strain was not reported.

References

1. Aguilera, L.; Toloza, L.; Giménez, R.; Odena, A.; Oliveira, E.; Aguilar, J.; Badia, J.; Baldomà, L. Proteomic Analysis of Outer Membrane Vesicles from the Probiotic Strain *Escherichia coli* Nissle 1917. *Proteomics* **2014**, *14*, 222–229.
2. Fábrega, M.J.; Aguilera, L.; Giménez, R.; Varela, E.; Cañas, M.A.; Antolín, M.; Badía, J.; Baldomà, L. Activation of Immune and Defense Responses in the Intestinal Mucosa by Outer Membrane Vesicles of Commensal and Probiotic *Escherichia coli* Strains. *Front. Microbiol*. **2016**, *7*, 705.
3. Fábrega, M.J.; Rodríguez-Nogales, A.; Garrido-Mesa, J.; Algieri, F.; Badía, J.; Giménez, R.; Gálvez, J.; Baldomà, L. Intestinal Anti-inflammatory Effects of Outer Membrane Vesicles from *Escherichia coli* Nissle 1917 in DSS-Experimental Colitis in Mice. *Front. Microbiol*. **2017**, *8*, 1274.
4. Pérez-Cruz, C.; Cañas, M.A.; Giménez, R.; Badia, J.; Mercade, E.; Baldomà, L.; Aguilera, L. Membrane Vesicles Released by a Hypervesiculating *Escherichia coli* Nissle 1917 *tolR* Mutant Are Highly Heterogeneous and Show Reduced Capacity for Epithelial Cell Interaction and Entry. *PLoS One* **2016**, *11*, e0169186.
5. Hirayama, S.; Nakao, R. Glycine Significantly Enhances Bacterial Membrane Vesicle Production: A Powerful Approach for Isolation of LPS-Reduced Membrane Vesicles of Probiotic *Escherichia coli*. *Microb. Biotechnol*. **2020**, *13*, 1162–1178.
6. Hu, R.; Lin, H.; Li, J.; Zhao, Y.; Wang, M.; Sun, X.; Min, Y.; Gao, Y.; Yang, M. Probiotic *Escherichia coli* Nissle 1917-Derived Outer Membrane Vesicles Enhance Immunomodulation and Antimicrobial Activity in RAW264.7 Macrophages. *BMC Microbiol*. **2020**, *20*, 268.
7. Hong, J.; Dauros-Singorenko, P.; Whitcombe, A.; Payne, L.; Blenkiron, C.; Phillips, A.; Swift, S. Analysis of the *Escherichia coli* Extracellular Vesicle Proteome Identifies Markers of Purity and Culture Conditions. *J. Extracell. Vesicles* **2019**, *8*, 1632099.
8. Ashrafian, F.; Shahriary, A.; Behrouzi, A.; Moradi, H.R.; Keshavarz Azizi Raftar, S.; Lari, A.; Hadifar, S.; Yaghoubfar, R.; Ahmadi Badi, S.; Khatami, S.; et al. *Akkermansia muciniphila*-Derived Extracellular Vesicles as a Mucosal Delivery Vector for Amelioration of Obesity in Mice. *Front. Microbiol*. **2019**, *10*, 2155.
9. Ashrafian, F.; Behrouzi, A.; Shahriary, A.; Badi, S.A.; Davari, M.; Khatami, S.; Jamnani, F.R.; Fateh, A.; Vaziri, F.; Siadat, S.D. Comparative Study of Effect of *Akkermansia muciniphila* and Its Extracellular Vesicles on Toll-Like Receptors and Tight Junction. *Gastroenterol. Hepatol. From Bed to Bench* **2019**, *12*, 168.
10. Ashrafian, F.; Keshavarz Azizi Raftar, S.; Lari, A.; Shahryari, A.; Abdollahiyan, S.; Moradi, H.R.; Masoumi, M.; Davari, M.; khatami, S.; Omrani, M.D.; et al. Extracellular Vesicles and Pasteurized Cells Derived from *Akkermansia muciniphila* Protect against High-Fat Induced Obesity in Mice. *Microb. Cell Fact*. **2021**, *20*, 219.

11. Keshavarz Azizi Raftar, S.; Ashrafian, F.; Yadegar, A.; Lari, A.; Moradi, H.R.; Shahriary, A.; Azimirad, M.; Alavifard, H.; Mohsenifar, Z.; Davari, M.; et al. The Protective Effects of Live and Pasteurized *Akkermansia muciniphila* and Its Extracellular Vesicles against HFD/CCl4-Induced Liver Injury. *Microbiol. Spectr*. **2021**, *9*, e0048421.
12. Chelakkot, C.; Choi, Y.; Kim, D.K.; Park, H.T.; Ghim, J.; Kwon, Y.; Jeon, J.; Kim, M.S.; Jee, Y.K.; Gho, Y.S.; et al. *Akkermansia muciniphila*-Derived Extracellular Vesicles Influence Gut Permeability through the Regulation of Tight Junctions. *Exp. Mol. Med*. **2018**, *50*, e450–e450.
13. Luo, Z.W.; Xia, K.; Liu, Y.W.; Liu, J.H.; Rao, S.S.; Hu, X.K.; Chen, C.Y.; Xu, R.; Wang, Z.X.; Xie, H. Extracellular Vesicles from *Akkermansia muciniphila* Elicit Antitumor Immunity Against Prostate Cancer via Modulation of CD8 + T Cells and Macrophages. *Int. J. Nanomedicine* **2021**, *16*, 2949–2963.
14. Yaghoubfar, R.; Behrouzi, A.; Zare Banadkoki, E.; Ashrafian, F.; Lari, A.; Vaziri, F.; Nojoumi, S.A.; Fateh, A.; Khatami, S.; Siadat, S.D. Effect of *Akkermansia muciniphila, Faecalibacterium prausnitzii*, and Their Extracellular Vesicles on the Serotonin System in Intestinal Epithelial Cells. *Probiotics Antimicrob. Proteins* **2021**, *13*, 1546–1556.
15. Ghaderi, F.; Sotoodehnejadnematalahi, F.; Hajebrahimi, Z.; Fateh, A.; Seyed, &; Siadat, D. Effects of Active, Inactive, and Derivatives of *Akkermansia muciniphila* on The Expression of The Endocannabinoid System and PPARs Genes. *Sci. Rep*. **2022**, *12*, 10031.
16. Bajic, S.S.; Cañas, M.A.; Tolinacki, M.; Badia, J.; Sánchez, B.; Golic, N.; Margolles, A.; Baldomá, L.; Ruas-Madiedo, P. Proteomic Profile of Extracellular Vesicles Released by *Lactiplantibacillus plantarum* BGAN8 and Their Internalization by Non-Polarized HT29 Cell Line. *Sci. Rep*. **2020**, *10*, 21829.
17. Hao, H.; Zhang, X.; Tong, L.; Liu, Q.; Liang, X.; Bu, Y.; Gong, P.; Liu, T.; Zhang, L.; Xia, Y.; et al. Effect of Extracellular Vesicles Derived From *Lactobacillus plantarum* Q7 on Gut Microbiota and Ulcerative Colitis in Mice. *Front. Immunol*. **2021**, *12*, 5167.
18. Kim, M.H.; Choi, S.J.; Choi, H. Il; Choi, J.P.; Park, H.K.; Kim, E.K.; Kim, M.J.; Moon, B.S.; Min, T.K.; Rho, M.; et al. *Lactobacillus plantarum*-Derived Extracellular Vesicles Protect Atopic Dermatitis Induced by *Staphylococcus aureus*-Derived Extracellular Vesicles. *Allergy. Asthma Immunol. Res*. **2018**, *10*, 516–532.
19. Lee, B.H.; Wu, S.C.; Shen, T.L.; Hsu, Y.Y.; Chen, C.H.; Hsu, W.H. The Applications of *Lactobacillus plantarum*-Derived Extracellular Vesicles as a Novel Natural Antibacterial Agent for Improving Quality and Safety in Tuna Fish. *Food Chem*. **2021**, *340*, 128104.
20. Liu, H.; Zhao, F.; Zhang, K.; Zhao, J.; Wang, Y. Investigating the Growth Performance, Meat Quality, Immune Function and Proteomic Profiles of Plasmal Exosomes in *Lactobacillus plantarum*-Treated Broilers with Immunological Stress. *Food Func*t. **2021**, *12*, 11790–11807.
21. Müller, L.; Kuhn, T.; Koch, M.; Fuhrmann, G. Stimulation of Probiotic Bacteria Induces Release of Membrane Vesicles with Augmented Anti-inflammatory Activity. *ACS Appl. Bio Mater*. **2021**, *4*, 3739–3748.
22. Kuhn, T.; Koch, M.; Fuhrmann, G.; Kuhn, T.; Fuhrmann, G.; Koch, M. Probiomimetics—Novel *Lactobacillus*-Mimicking Microparticles Show Anti-Inflammatory and Barrier-Protecting Effects in Gastrointestinal Models. *Small* **2020**, *16*, 2003158.
23. Li, M.; Lee, K.; Hsu, M.; Nau, G.; Mylonakis, E.; Ramratnam, B. *Lactobacillus*-Derived Extracellular Vesicles Enhance Host Immune Responses against Vancomycin-Resistant Enterococci. *BMC Microbiol*. **2017**, *17*, 66.
24. Morishita, M.; Sagayama, R.; Yamawaki, Y.; Yamaguchi, M.; Katsumi, H.; Yamamoto, A. Activation of Host Immune Cells by Probiotic-Derived Extracellular Vesicles via TLR2-Mediated Signaling Pathways. *Biol. Pharm. Bull*. **2022**, *45*, 354–359.
25. Morishita, M.; Horita, M.; Higuchi, A.; Marui, M.; Katsumi, H.; Yamamoto, A. Characterizing Different Probiotic-Derived Extracellular Vesicles as a Novel Adjuvant for Immunotherapy. *Mol. Pharm*. **2021**, *18*, 1080–1092.
26. Kim, H.; Kim, M.; Myoung, K.; Kim, W.; Ko, J.; Kim, K.P.; Cho, E.G. Comparative Lipidomic Analysis of Extracellular Vesicles Derived from *Lactobacillus plantarum* APsulloc 331261 Living in Green Tea Leaves Using Liquid Chromatography-Mass Spectrometry. *Int. J. Mol. Sci*. **2020**, *21*, 8076.
27. Kim, W.; Lee, E.J.; Bae, I.H.; Myoung, K.; Kim, S.T.; Park, P.J.; Lee, K.H.; Pham, A.V.Q.; Ko, J.; Oh, S.H.; et al. *Lactobacillus plantarum*-Derived Extracellular Vesicles Induce Anti-Inflammatory M2 Macrophage Polarization *In Vitro. J. Extracell. Vesicles* **2020**, *9*, 1793514.

28. Yang, Z.; Gao, Z.; Yang, Z.; Zhang, Y.; Chen, H.; Yang, X.; Fang, X.; Zhu, Y.; Zhang, J.; Ouyang, F.; et al. *Lactobacillus plantarum*-Derived Extracellular Vesicles Protect against Ischemic Brain Injury via The MicroRNA-101a-3p/c-Fos/TGF-β Axis. *Pharmacol. Res*. **2022**, *182*, 106332.
29. Jo, C.S.; Myung, C.H.; Yoon, Y.C.; Ahn, B.H.; Min, J.W.; Seo, W.S.; Lee, D.H.; Kang, H.C.; Heo, Y.H.; Choi, H.; et al. The Effect of *Lactobacillus plantarum* Extracellular Vesicles from Korean Women in Their 20s on Skin Aging. *Curr. Issues Mol. Biol*. **2022**, *44*, 526–540.
30. Bäuerl, C.; Coll-Marqués, J.M.; Tarazona-González, C.; Pérez-Martínez, G. *Lactobacillus casei* Extracellular Vesicles Stimulate EGFR Pathway Likely Due to the Presence of Proteins P40 and P75 Bound to Their Surface. *Sci. Rep*. **2020**, *10*, 19237.
31. Rubio, A.P.D.; Martínez, J.H.; Casillas, D.C.M.; Leskow, F.C.; Piuri, M.; Pérez, O.E. *Lactobacillus casei* BL23 Produces Microvesicles Carrying Proteins That Have Been Associated with Its Probiotic Effect. *Front. Microbiol*. **2017**, *8*, 1783.
32. Dean, S.N.; Leary, D.H.; Sullivan, C.J.; Oh, E.; Walper, S.A. Isolation and Characterization of *Lactobacillus*-Derived Membrane Vesicles. *Sci. Rep*. **2019**, *9*, 877.
33. Vargoorani, M.E.; Modarressi, M.H.; Vaziri, F.; Motevaseli, E.; Siadat, S.D. Stimulatory Effects of *Lactobacillus casei* Derived Extracellular Vesicles on Toll-Like Receptor 9 Gene Expression and Cytokine Profile in Human Intestinal Epithelial Cells. *J. Diabetes Metab. Disord*. **2020**, *19*, 223–231.
34. Behzadi, E.; Mahmoodzadeh Hosseini, H.; Imani Fooladi, A.A. The Inhibitory Impacts of *Lactobacillus rhamnosus* GG-Derived Extracellular Vesicles on the Growth of Hepatic Cancer Cells. *Microb. Pathog*. **2017**, *110*, 1–6.
35. Tong, L.; Zhang, X.; Hao, H.; Liu, Q.; Zhou, Z.; Liang, X.; Liu, T.; Gong, P.; Zhang, L.; Zhai, Z.; et al. *Lactobacillus rhamnosus* GG Derived Extracellular Vesicles Modulate Gut Microbiota and Attenuate Inflammatory in DSS-Induced Colitis Mice. *Nutrients* **2021**, *13*, 3319.
36. Keyhani, G.; Hosseini, H.M.; Salimi, A. Effect of Extracellular Vesicles of *Lactobacillus rhamnosus* GG on The Expression of CEA Gene and Protein Released by Colorectal Cancer Cells. *Iran. J. Microbiol*. **2022**, *14*, 90–96.
37. Champagne-Jorgensen, K.; Mian, M.F.; McVey Neufeld, K.A.; Stanisz, A.M.; Bienenstock, J. Membrane Vesicles of *Lacticaseibacillus rhamnosus* JB-1 Contain Immunomodulatory Lipoteichoic Acid and Are Endocytosed by Intestinal Epithelial Cells. *Sci. Rep*. **2021**, *11*, 13756.
38. Champagne-Jorgensen, K.; Jose, T.A.; Stanisz, A.M.; Mian, M.F.; Hynes, A.P.; Bienenstock, J. Bacterial Membrane Vesicles and Phages in Blood After Consumption of *Lacticaseibacillus rhamnosus* JB-1. *Gut Microbes* **2021**, *13*, e1993583.
39. Shi, Y.; Meng, L.; Zhang, C.; Zhang, F.; Fang, Y. Extracellular Vesicles of *Lacticaseibacillus paracasei* PC-H1 Induce Colorectal Cancer Cells Apoptosis via PDK1/AKT/Bcl-2 Signaling Pathway. *Microbiol. Res*. **2021**, *255*, 126921.
40. Choi, J.H.; Moon, C.M.; Shin, T.S.; Kim, E.K.; McDowell, A.; Jo, M.K.; Joo, Y.H.; Kim, S.E.; Jung, H.K.; Shim, K.N.; et al. *Lactobacillus paracasei*-Derived Extracellular Vesicles Attenuate the Intestinal Inflammatory Response by Augmenting the Endoplasmic Reticulum Stress Pathway. *Exp. Mol. Med*. **2020**, *52*, 423–437.
41. Shiraishi, T.; Yokota, S.; Sato, Y.; Ito, T.; Fukiya, S.; Yamamoto, S.; Sato, T.; Yokota, A. Lipoteichoic Acids are Embedded in Cell Walls during Logarithmic Phase, but Exposed on Membrane Vesicles in *Lactobacillus gasseri* JCM 1131^T. *Benef. Microbes* **2018**, *9*, 653–662.
42. Ñahui Palomino, R.A.; Vanpouille, C.; Laghi, L.; Parolin, C.; Melikov, K.; Backlund, P.; Vitali, B.; Margolis, L. Extracellular Vesicles from Symbiotic Vaginal Lactobacilli Inhibit HIV-1 Infection of Human Tissues. *Nat. Commun*. **2019**, *10*, 5656.
43. Harrison, N.A.; Gardner, C.L.; da Silva, D.R.; Gonzalez, C.F.; Lorca, G.L. Identification of Biomarkers for Systemic Distribution of Nanovesicles From *Lactobacillus johnsonii* N6.2. *Front. Immunol*. **2021**, *12*, 3491.
44. Grande, R.; Celia, C.; Mincione, G.; Stringaro, A.; Di Marzio, L.; Colone, M.; Di Marcantonio, M.C.; Savino, L.; Puca, V.; Santoliquido, R.; et al. Detection and Physicochemical Characterization of Membrane Vesicles (MVs) of *Lactobacillus reuteri* DSM 17938. *Front. Microbiol*. **2017**, *8*, 1040.

45. Puca, V.; Ercolino, E.; Celia, C.; Bologna, G.; Di Marzio, L.; Mincione, G.; Marchisio, M.; Miscia, S.; Muraro, R.; Lanuti, P.; et al. Detection and Quantification of eDNA-Associated Bacterial Membrane Vesicles by Flow Cytometry. *Int. J. Mol. Sci*. **2019**, *20*, 5307.
46. West, C.L.; Stanisz, A.M.; Mao, Y.K.; Champagne-Jorgensen, K.; Bienenstock, J.; Kunze, W.A. Microvesicles from *Lactobacillus reuteri* (DSM-17938) Completely Reproduce Modulation of Gut Motility by Bacteria in Mice. *PLoS One* **2020**, *15*, e0225481.
47. Hu, R.; Lin, H.; Wang, M.; Zhao, Y.; Liu, H.; Min, Y.; Yang, X.; Gao, Y.; Yang, M. *Lactobacillus reuteri*-Derived Extracellular Vesicles Maintain Intestinal Immune Homeostasis against Lipopolysaccharide-Induced Inflammatory Responses in Broilers. *J. Anim. Sci. Biotechnol*. **2021**, *12*, 25.
48. Seo, M.K.; Park, E.J.; Ko, S.Y.; Choi, E.W.; Kim, S. Therapeutic Effects of Kefir Grain *Lactobacillus*-Derived Extracellular Vesicles in Mice with 2,4,6-Trinitrobenzene Sulfonic Acid-Induced Inflammatory Bowel Disease. *J. Dairy Sci*. **2018**, *101*, 8662–8671.
49. Yamasaki-Yashiki, S.; Miyoshi, Y.; Nakayama, T.; Kunisawa, J.; Katakura, Y. IgA-Enhancing Effects of Membrane Vesicles Derived from *Lactobacillus sakei* subsp. *sakei* NBRC15893. *Biosci. Microbiota, Food Heal*. **2019**, *38*, 29.
50. Chen, C.Y.; Rao, S.S.; Yue, T.; Tan, Y.J.; Yin, H.; Chen, L.J.; Luo, M.J.; Wang, Z.; Wang, Y.Y.; Hong, C.G.; et al. Glucocorticoid-Induced Loss of Beneficial Gut Bacterial Extracellular Vesicles Is Associated with The Pathogenesis of Osteonecrosis. *Sci. Adv*. **2022**, *8*, eabg8335.
51. Yoon, Y.C.; Ahn, B.H.; Min, J.W.; Lee, K.R.; Park, S.H.; Kang, H.C. Stimulatory Effects of Extracellular Vesicles Derived from *Leuconostoc holzapfelii* That Exists in Human Scalp on Hair Growth in Human Follicle Dermal Papilla Cells. *Curr. Issues Mol. Biol*. **2022**, *44*, 845–866.
52. Alpdundar Bulut, E.; Bayyurt Kocabas, B.; Yazar, V.; Aykut, G.; Guler, U.; Salih, B.; Surucu Yilmaz, N.; Ayanoglu, I.C.; Polat, M.M.; Akcali, K.C.; et al. Human Gut Commensal Membrane Vesicles Modulate Inflammation by Generating M2-like Macrophages and Myeloid-Derived Suppressor Cells. *J. Immunol*. **2020**, *205*, 2707–2718.
53. Liu, Y.; Tempelaars, M.H.; Boeren, S.; Alexeeva, S.; Smid, E.J.; Abee, T. Extracellular Vesicle Formation in *Lactococcus lactis* is Stimulated by Prophage-Encoded Holin–Lysin System. *Microb. Biotechnol*. **2022**, *15*, 1281–1295.
54. Lee, D.H.; Park, H.K.; Lee, H.R.; Sohn, H.; Sim, S.; Park, H.J.; Shin, Y.S.; Kim, Y.K.; Choi, Y.; Park, H.S. Immunoregulatory Effects of *Lactococcus lactis*-Derived Extracellular Vesicles in Allergic Asthma. *Clin. Transl. Allergy* **2022**, *12*, e12138.
55. Nishiyama, K.; Takaki, T.; Sugiyama, M.; Fukuda, I.; Aiso, M.; Mukai, T.; Odamaki, T.; Xiao, J. zhong; Osawa, R.; Okada, N. Extracellular Vesicles Produced by *Bifidobacterium longum* Export Mucin-Binding Proteins. *Appl. Environ. Microbiol*. **2020**, *86*, e01464-20.
56. Rodovalho, V. de R.; Luz, B.S.R. da; Rabah, H.; do Carmo, F.L.R.; Folador, E.L.; Nicolas, A.; Jardin, J.; Briard-Bion, V.; Blottière, H.; Lapaque, N.; et al. Extracellular Vesicles Produced by the Probiotic *Propionibacterium freudenreichii* CIRM-BIA 129 Mitigate Inflammation by Modulating the NF-κB Pathway. *Front. Microbiol*. **2020**, *11*, 1544.
57. de Rezende Rodovalho, V.; da Luz, B.S.R.; Nicolas, A.; do Carmo, F.L.R.; Jardin, J.; Briard-Bion, V.; Jan, G.; Loir, Y. Le; de Carvalho Azevedo, V.A.; Guédon, E. Environmental Conditions Modulate the Protein Content and Immunomodulatory Activity of Extracellular Vesicles Produced by the Probiotic *Propionibacterium freudenreichii. Appl. Environ. Microbiol*. **2020**, *87*, e02263-20.
58. Rubio, A.P.D.; Martínez, J.; Palavecino, M.; Fuentes, F.; López, C.M.S.; Marcilla, A.; Pérez, O.E.; Piuri, M. Transcytosis of *Bacillus subtilis* Extracellular Vesicles through an *In Vitro* Intestinal Epithelial Cell Model. *Sci. Rep*. **2020**, *10*, 3120.
59. Ma, L.; Shen, Q.; Lyu, W.; Lv, L.; Wang, W.; Yu, M.; Yang, H.; Tao, S.; Xiao, Y. *Clostridium butyricum* and Its Derived Extracellular Vesicles Modulate Gut Homeostasis and Ameliorate Acute Experimental Colitis. *Microbiol. Spectr*. **2022**, *10*, e0136822.
60. Liang, L.; Yang, C.; Liu, L.; Mai, G.; Li, H.; Wu, L.; Jin, M.; Chen, Y. Commensal Bacteria-Derived Extracellular Vesicles Suppress Ulcerative Colitis through Regulating The Macrophages Polarization and Remodeling The Gut Microbiota. *Microb. Cell Fact*. **2022**, *21*, 88.
